# Modulating Thermal Properties of Polymers through Crystal Engineering

**DOI:** 10.1002/anie.202212688

**Published:** 2023-02-01

**Authors:** Luzia S. Germann, Elvio Carlino, Antonietta Taurino, Oxana V. Magdysyuk, Dario Voinovich, Robert E. Dinnebier, Dejan‐Krešimir Bučar, Dritan Hasa

**Affiliations:** ^1^ Max Planck Institute for Solid State Research Heisenberg Straße 1 70569 Stuttgart Germany; ^2^ Istituto di Cristallografia—Consiglio Nazionale delle Ricerche (IC—CNR) Via Amendola 122/O 70126 Bari Italy; ^3^ Institute for Microelectronics and Microsystems, Consiglio Nazionale delle Ricerche (IMM—CNR) Via Monteroni 73100 Lecce Italy; ^4^ Diamond Light Source Ltd. Harwell Science and Innovation Campus Didcot OX11 0DE UK; ^5^ Department of Chemical and Pharmaceutical Sciences University of Trieste Via Giorgieri 1 34127 Trieste Italy; ^6^ Department of Chemistry University College London 20 Gordon Street London WC1H 0AJ UK

**Keywords:** Cocrystals, Crystal Engineering, Mechanochemistry, Polymers, Solid Solutions

## Abstract

Crystal engineering has exclusively focused on the development of advanced materials based on small organic molecules. We now demonstrate how the cocrystallization of a polymer yields a material with significantly enhanced thermal stability but equivalent mechanical flexibility. Isomorphous replacement of one of the cocrystal components enables the formation of solid solutions with melting points that can be readily fine‐tuned over a usefully wide temperature range. The results of this study credibly extend the scope of crystal engineering and cocrystallization from small molecules to polymers.

## Graphical abstract

Crystal engineering of multicomponent solids involving both small and polymeric molecules resulted in the preparation of cocrystals with melting points higher than the melting point of the pure polymer. At the same time, the high mechanical flexibility of the polymer is maintained even under harsh milling conditions, allowing the formation of toroidal nanoparticles with small inner radii and high curvatures.

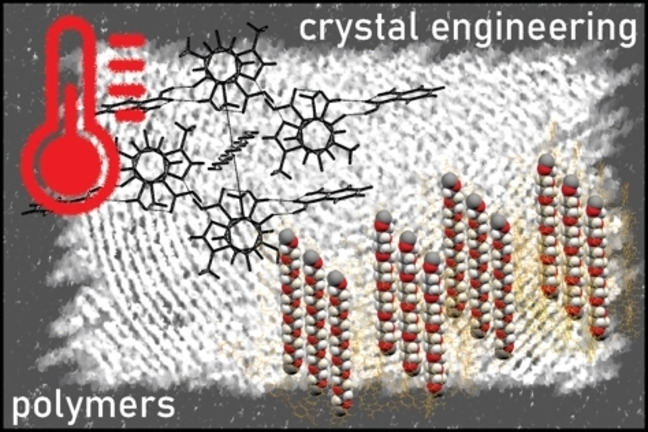

## Introduction

Crystal engineering concerns the design and the synthesis of functional solids with targeted properties.[^1^, ^2^, ^3^] For over thirty years, this maturing area of solid‐state chemistry[^4^, ^5^, ^6^] has been solely focused on solids comprised of small molecules, particularly specialty chemicals (e.g. pharmaceuticals,^[7]^ energetic materials,^[8]^ pigments,^[9]^ agrochemicals,^[10]^ optical^[11]^ and electronic materials^[12]^). Small molecules have also been used as metal ligands and building blocks to engineer porous materials^[13]^ (e.g. metal‐organic structures, covalent organic frameworks and porous molecular crystals) with applications in catalysis, imaging and sensing, gas separation, carbon capture and other applications.[^14^, ^15^] We now report the results of a study that feasibly expands the scope of crystal engineering from small molecules to polymers by showing how thermal properties of polyethylene glycol (PEG)—a ubiquitous polymer with applications in medicine, biology, the chemical industry, consumer healthcare and the entertainment industry—can be fine‐tuned through cocrystallization and other well‐established crystal engineering strategies. We note that crystal engineering was previously linked to polymer chemistry, either through the synthesis of polymers in topotactic solid‐state polymerizations,^[16]^ through an unexpected cocrystallization of a PEG excipient with pharmaceuticals^[17]^ and inorganics,^[18]^ as well as through particle engineering using DNA.^[19]^ This study, however, represents to our knowledge the first case wherein physicochemical properties of a polymer were enhanced and rationally modulated through cocrystallization and the modification of a polymer crystal structure.

## Results and Discussion

**Scheme 1:**
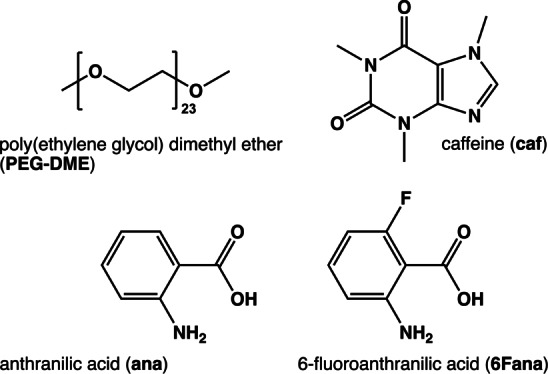
Chemical structures of poly(ethylene glycol) dimethyl ether (**PEG‐DME**, *M*_n_≈1000 g mol^−1^), caffeine (**caf**), anthranilic acid (**ana**) and 6‐fluoroanthranilic acid (**6Fana)**.

Our recent focus on polymer‐assisted grinding (POLAG) of pharmaceuticals^[20]^ and in situ monitoring of mechanochemical reactions^[21]^ through powder X‐ray diffraction (PXRD), led to the unexpected discovery of a cocrystal composed of caffeine (**caf**), anthranilic acid (**ana**) and a PEG polymer (see Supporting Information document). Subsequent efforts to structurally characterize these cocrystals led to the preparation and structure determination of two related cocrystals. The first cocrystal, being composed of poly(ethylene glycol) dimethyl ether (*M*_n_≈1000 g mol^−1^, **PEG‐DME**), **caf** and **ana** (Scheme 1), was produced unexpectedly, while the second one, comprised of **PEG‐DME**, **caf** and 6‐fluoro‐anthranilic acid (**6Fana**), was pursued following crystal design strategies.

Both solids, (**PEG‐DME**) ⋅ (**caf**)_23_ ⋅ (**ana**)_46_, hereafter named (**1**), and (**PEG‐DME**) ⋅ (**caf**)_23_ ⋅ (**6Fana**)_46_, henceforth named (**2**), were analyzed using a combination of PXRD, single crystal X‐ray diffraction^[22]^ (SCXRD) and differential‐scanning calorimetry (DSC), while an additional morphological and structural characterization of **1** was accomplished using low dose atomic resolution transmission electron microscopy (Holo‐TEM) imaging^[23]^ and through the analyses of relevant diffractograms.

We demonstrate that cocrystallization of **PEG‐DME** with **caf** and **ana** significantly boosts its thermal stability from 36 °C to 98 °C *without compromising the mechanical flexibility of the polymer*. We also show how SCXRD studies guided the isomorphous replacement of the **ana** cocrystal component with **6Fana** to further boost the melting point of the polymer crystal form to 128 °C, while the formation of solid solutions allowed the fine‐tuning of polymer formulation melting points over a temperature range from 98 °C to 128 °C. And while we acknowledge that the discovery of **1** was serendipitous, rather than targeted and guided by crystal design principles, we assert the relevance of our findings, as they demonstrate that even polymers composed of functional groups that are exceedingly difficult to engage in hydrogen bonding (such as the ether moieties in PEG), are indeed susceptible to the improvement of their physicochemical solid‐state properties through cocrystallization and crystal engineering.

**Figure 1:**
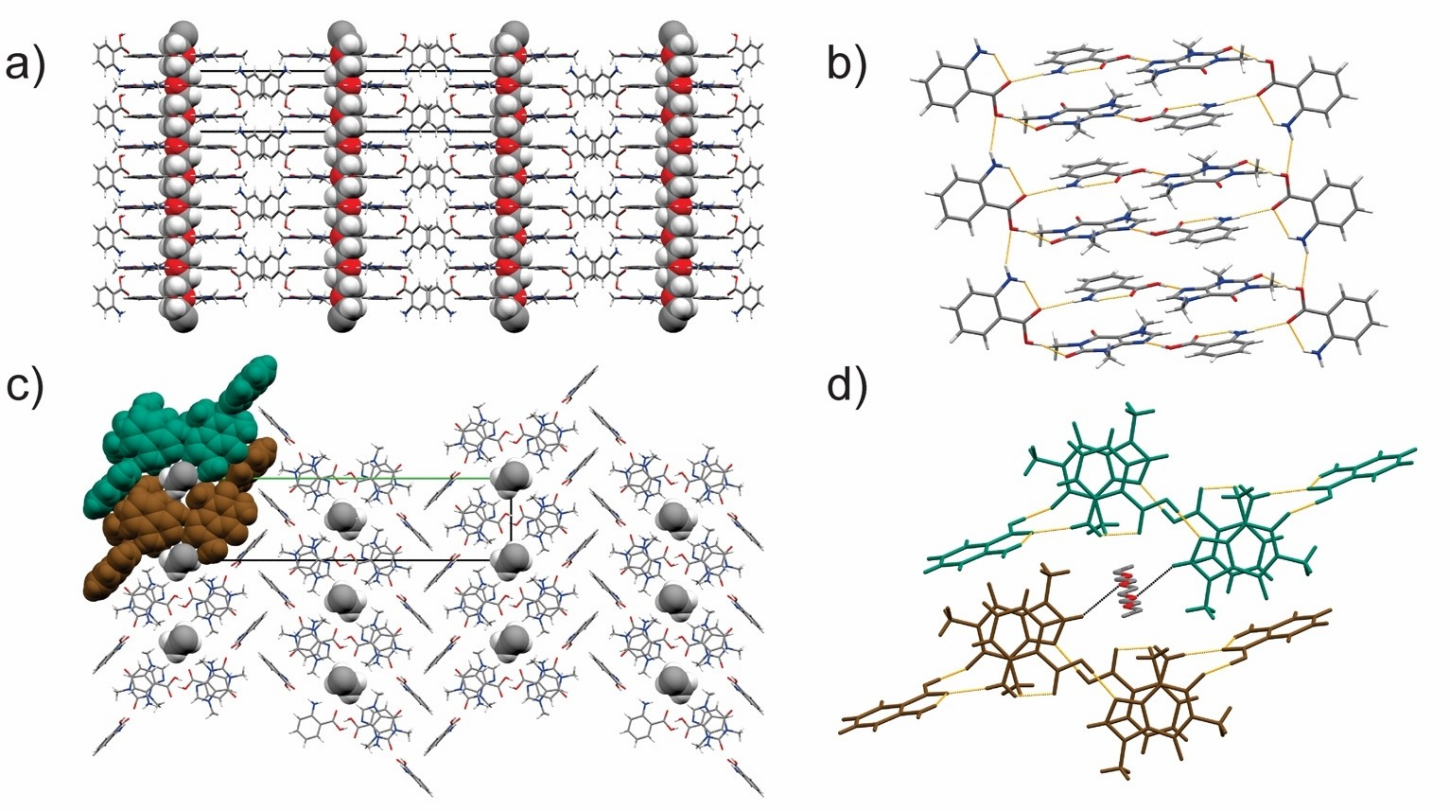
X‐ray crystal structure of **1** showing: a) **PEG‐DME** molecules (shown using the spacefill style) running parallel to the crystallographic *a*‐axis, b) the hydrogen bonding in the **caf** : **ana** molecular ladder (hydrogen bonds are shown in orange), c) two **caf** : **ana** ladder assemblies (shown in brown and green) surrounding **PEG‐DME** and d) C−H(**caf**)⋅⋅⋅O(**PEG‐DME**) interactions (shown in black) between the imidazole groups of **caf** and the ether group of **PEG‐DME** (for clarity shown without hydrogen atoms). Color scheme for atom types: grey—carbon, white—hydrogen, blue–nitrogen, red—oxygen.

Both POLAG and melt crystallization of **PEG‐DME**, **caf** and **ana** in a 1 : 23 : 46 ratio afford the formation of **1** in the absence of any solvents. SCXRD studies of a batch of **1**, obtained through melt co‐crystallization, revealed that **PEG‐DME**, **caf** and **ana** cocrystallize in the monoclinic space group *P*2_1_/*c* with a 1/46 fraction of **PEG‐DME**, one half molecule of **caf** and two half molecules of **ana** in the asymmetric unit.^[24]^ The fully ordered polymer adopts a linear conformation and is positioned parallel to the crystallographic *a*‐axis (Figure 1a).[^25^, ^26^, ^27^] Each **PEG‐DME** molecule is fully surrounded by two supramolecular ladders^[28]^ formed from **caf** and **ana** molecules in a 1 : 2 ratio (Figures 1b,c). The ladders are composed of two coplanar **caf** and **ana** molecules acting as ladder rungs, which are hydrogen‐bonded to **ana** molecules operating as ladder side rails (Figures 1c). The **ana** side rails assume a nearly orthogonal orientation to the coplanar **caf** : **ana** rungs and are disordered over two positions (occupancies: 0.95 : 0.05). The **ana** side‐rail constituents are connected through N−H(**ana**)⋅⋅⋅O(**ana**) hydrogen bonds [*d*(O⋅⋅⋅N)=2.96 Å]. The planar **caf** : **ana** rungs are held together by an $R_{2}^{2}\left(8\right)$ carboxylic‐acid : imidazole synthon, which is dominated by an O−H(**ana**)⋅⋅⋅N(**caf**) hydrogen bond [*d*(O⋅⋅⋅N)=2.71 Å]. The rungs are hydrogen‐bonded to the **ana** side rails of the molecular ladder through O−H(**ana**)⋅⋅⋅O(**caf**) and N−H(**ana**)⋅⋅⋅O(**ana**) and hydrogen bonds [*d*(O⋅⋅⋅N)=2.63 Å and *d*(O⋅⋅⋅N)=2.95 Å, respectively]. The **caf** : **ana** ladders interact with the polymer through a C−H(**caf**)⋅⋅⋅O(PEG) interaction between a **caf** imidazole groups and the ether group of **PEG‐DME** [*d*(C⋅⋅⋅O)=3.43 Å] (Figure 1d). The position of the terminal **PEG‐DME** ether functional groups could not be determined owing to their low weight percentage in the polymer.

Thermal analyses of **1** revealed that the cocrystallization of **PEG‐DME** yields a solid with significantly improved thermal stability, increasing the melting point from 36 °C (for **PEG‐DME**) to 98 °C. The increase in thermal stability was attributed to the incorporation of **PEG‐DME** into a thermally stable, strongly hydrogen‐bonded molecular scaffold built from **caf** and **ana**. We note that polymorphs of the binary (**caf**) ⋅ (**ana**) cocrystal, featuring structures that are sustained by analogous hydrogen bonds, melt in the 100–110 °C temperature range.^[29]^ The increased thermal stability of cocrystallized **PEG‐DME** prompted us to explore the possibility of further tuning the thermal properties of **1** through modifications to its crystal structure; more specifically, through the formation of organic solid solutions,[^30^, ^31^] otherwise also known as organic alloys^[32]^ and mixed molecular crystals.^[33]^ Following Kitaigorodskii's solubility principles,^[34]^ the working hypothesis of subsequent studies was that the design of another polymer cocrystal, isostructural to **1**, will enable the formation of a continuous solid solution by alloying of the two cocrystals in the entire compositional range.[^35^, ^36^] And this, in turn, is expected to permit further tuning of the thermal properties of **PEG‐DME**.

The formation of organic solid solutions is, according to Kitaigorodskii, only possible through the incorporation of molecular substituents (i.e., solutes) that are suitably similar in terms of size and shape to the main constituent of a molecular crystal.[^30^, ^34^] The extent to which two molecules are comparable is expressed by the coefficient of geometrical similarity (ϵ):

$$\epsilon =1-\;\frac{\Delta }{\tau }$$

(where *Δ* defines the minimal non‐overlapping volume of two molecules, while *τ* describes the maximal overlapping volume of both molecules).^[37]^ Kitaigorodskii further postulated that two molecules are miscible if the solute and the main constituent of a crystal exhibit an *ϵ* value greater than 0.80 and if the solute is not disrupting any hydrogen‐bond patterns in the crystal structure, or causing a destabilization of the crystal structure through unfavorable repulsive electrostatic interactions.^[34]^

**Figure 2:**
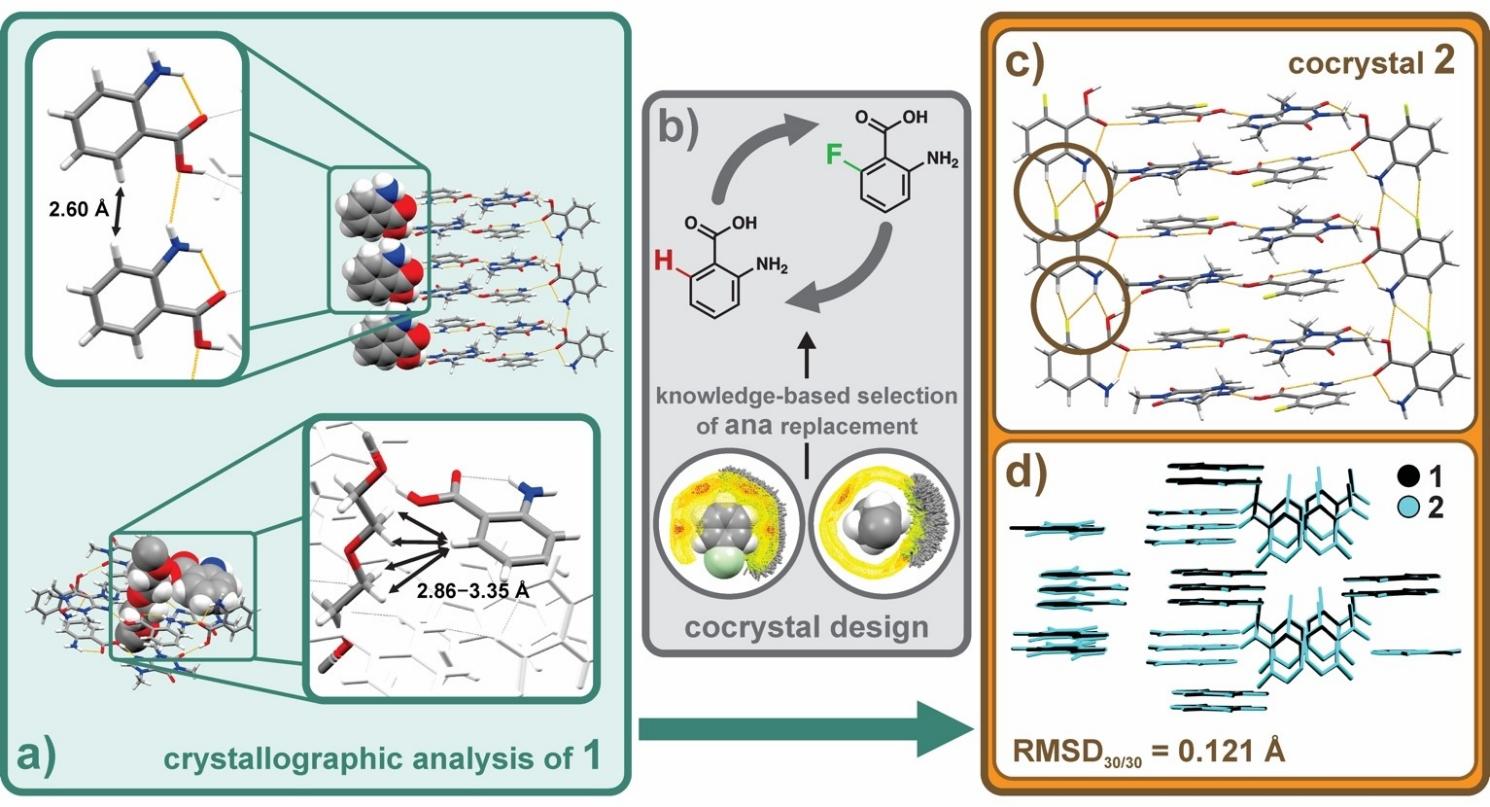
Crystal engineering of solid solution of polymers: a) crystallographic analyses of intermolecular distances between **PEG‐DME** and the unsubstituted *ortho*‐position of **ana** in the side rail of the supramolecular ladder in **1**, and between **PEG‐DME** and the *ortho*‐position of **ana** in the ladder rung; b) analyses of the *Cambridge Structural Database* (and its accompanying library of intermolecular interactions) led to the identification of **6Fana** as potential solute for the formation of solid solutions based on **1**; c) perspective view of the **caf** : **6Fana** molecular ladder in the mechanochemically prepared cocrystal **2**, highlighting the anticipated C−F(**6Fana**)⋅⋅⋅H(**6Fana**) interactions (hydrogen bonds are shown in orange); d) fully matched molecular positions in **1** and **2** (highlighted in black and cyan, respectively) through a crystal‐packing similarity analysis, underpinning the isostructural relationship of cocrystals **1** and **2**. Color scheme for a) and c): grey—carbon, white—hydrogen, blue—nitrogen, red—oxygen, yellow—fluorine orange—hydrogen bonds.

To narrow down the search for such suitable solute, a crystal structure analysis of **1** ensued. This inspection revealed that the unsubstituted *ortho* positions of both crystallographically independent **ana** molecules are not involved in any close intermolecular contacts. Specifically, one of the two *ortho*‐positioned **ana** hydrogen atoms is separated from a CH(**ana**) moiety at a distance of 2.60 Å (Figure 2a), while the other one is separated from a CH_2_(**PEG‐DME**) moiety by 2.86–3.35 Å (Figure 2a). Bearing in mind these considerable intermolecular distances, and the reasonably small size difference between hydrogen and fluorine atoms (van der Waals radii: 1.20 Å vs. 1.47 Å,^[38]^ respectively), it was concluded that the *ortho*‐positioned hydrogen atom in **ana** could be replaced with a fluorine atom (through the use of **6Fana**, Scheme 1) without causing any disruptions to the crystal structure; and hence enable the formation of an isomorphous crystal structure that is fully miscible with **1**.[^39^, ^40^] This surmise was further corroborated by an estimation of the geometrical similarity between **ana** and **6Fana**, and an analysis of spatial characteristics of C(sp^2^)−F⋅⋅⋅H−C(sp^2^) and C(sp^2^)−F⋅⋅⋅H−C(sp^3^) interactions. Specifically, the *ϵ* value for **ana** and **6Fana** (*ϵ*_**ana**/**6Fana**_=0.93) suggests that **6Fana** is fully miscible with **ana** in the structure of **1**, as it exceeds the required value of 0.80 and therefore satisfies Kitaigorodskii's basic rule of solubility.^[34]^ A survey of the Cambridge Structural Database^[41]^ was also performed using its *IsoStar* library of intermolecular interactions^[42]^ to estimate whether the geometry of relevant intermolecular C−H⋅⋅⋅H−C distances would meaningfully change upon a fluoro‐substitution of the *ortho*‐position in **ana**, and thereby become sterically disruptive. Our analyses suggested that the planned atom substitution could not only proceed without any distortion of the resulting crystal structure, but that it also feasibly places the fluorine atom in a position that could facilitate the emergence of attractive C−F(**6Fana**)⋅⋅⋅H(**6Fana**) and C−F(**6Fana**)⋅⋅⋅H(**PEG‐DME**) interactions, which are expected to further stabilize the structure of a related, isostructural polymer cocrystal.

As anticipated, a subsequent mechanochemical attempt to crystallize **PEG‐DME**, **caf** and **6Fana** in a 1 : 23 : 46 ratio resulted in the formation of **2**; a cocrystal that is isostructural to **1**. The crystal structure of **2** was determined by SCXRD after a suitable crystal was obtained through the slow evaporation of an ethyl acetate solution of **2**. The material crystallizes in the orthorhombic space group *Pna*2_1_ with a 1/23 fraction of **PEG‐DME**, one molecule of **caf** and two molecules of **6Fana** in the asymmetric unit (see Supporting Information document). The molecular ladder structure in **2** is analogous to that observed in **1**. The ladder rungs also feature the anticipated C−F(**6Fana**)⋅⋅⋅H(**6Fana**) (Figure 2c), while no short contacts were found between **PEG‐DME** and the **6Fana** molecules in the ladder steps.

The structural similarity of **1** and **2** was assessed by a combination of hydrogen‐bond pattern analyses and crystal‐packing‐similarity calculations (i.e. geometric analyses of molecular clusters using distance constraints to represent molecular packing[^43^, ^44^]) as implemented in *CCDC Mercury* ^[45]^ (see Supporting Information document). The isostructurality of the two polymer cocrystals was assessed following previously established criteria,[^46^, ^47^] and based on the observation that: 1) all molecules in the two molecular clusters (each comprised of 30 molecules representing the crystal structures) were successfully matched^[44]^ (RMSD_30_=0.121 Å), and 2) that the two solids display intermolecular interactions involving the same functional groups. Although the calculated unit‐cell similarity index^[43]^ (Π=0.0025) and the powder pattern similarity score (Σ=0.995) of **1** and **2** feature values that are consistent with those that were previously reported for isomorphous cocrystals,^[48]^ the pair of cocrystals was not classed as isomorphous owing to their discrepancy in crystal symmetry.^[43]^

**Figure 3:**
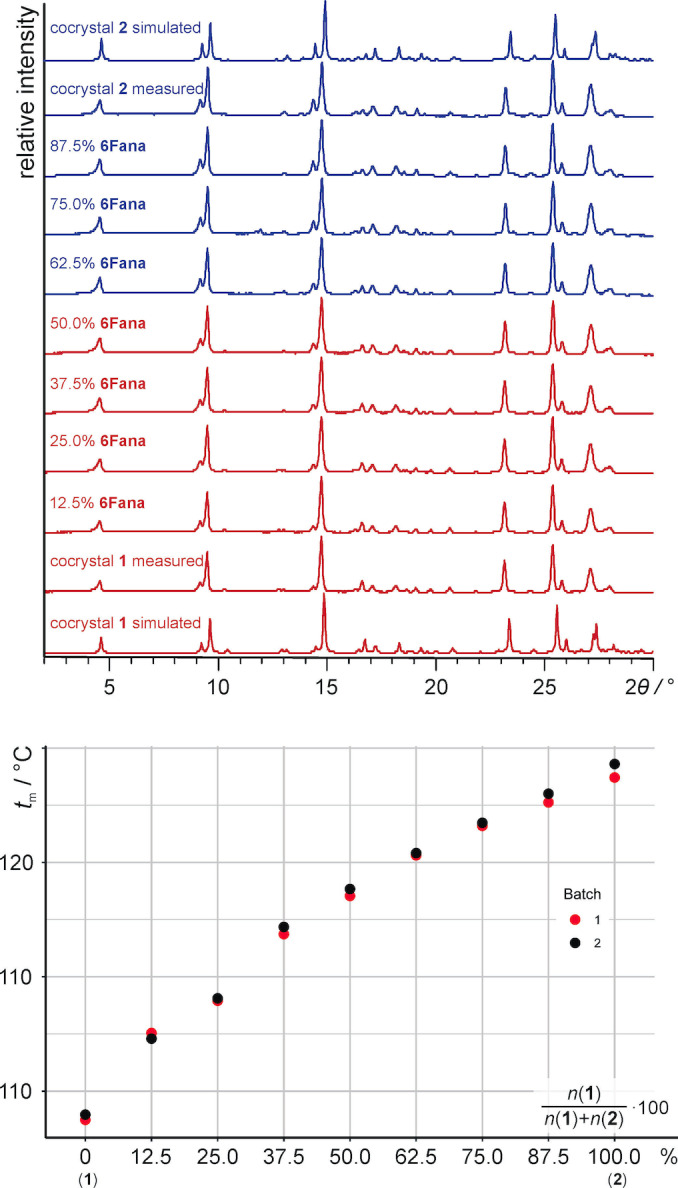
Diffractograms of cocrystals of **1**, **2** and solid solutions with varying ratios of **ana** and **6Fana** are shown in the upper graph. Solid solutions highlighted in red structurally resemble cocrystal **1**, while solid solutions highlighted in blue resemble **2**. The melting points of the solid solutions, as determined using differential‐scanning calorimetry, are shown in the lower graph.

Subsequent mechanochemical alloying of **1** and **2** showed that the two solids are fully miscible and resulted in the formation of a series of solid solutions with any relative ratios of **1** and **2** (Figure 3 and Section 4 in the Supporting Information document). Rietveld analyses showed that solid solutions containing up to 50 % **6Fana** are isomorphous with the structure of **1**, while those with more 50 % of **6Fana** adopt the structure of **2** (see Supporting Information document). DSC analyses of the seven prepared solid solutions (with relative amounts of **6Fana** varying from 12.5 % to 87.5 %) then revealed that the melting temperature of the **PEG‐DME** cocrystal can be fine‐tuned within the temperature range from 98 °C (the melting point of **1**) to 128 °C (the melting point of **2**) (Figure 3).

The enhanced thermal stability of cocrystallized **PEG‐DME** called for further investigations to determine whether the incorporation of **caf** and **ana** into the **PEG‐DME** solid‐state structure affected its mechanical flexibility. Low dose in‐line electron holography and low dose high‐resolution TEM (HRTEM) imaging of **1** was pursued to understand directly its morphological and structural properties at atomic resolution. The very high sensitivity of **1** to radiation damage required the use of tailored sample‐preparation methods and low dose imaging approaches based on in‐line holography surveys^[23]^ capable of producing holograms with a satisfying signal to noise ratio by using electron densities as low as 0.2 e^−^ Å^−2^. The low dose HRTEM imaging was pursued in combination with relevant diffraction measurements to study a powdered specimen. The atomic resolution images and the relevant diffractograms have been successfully simulated and indexed by the *Java Electron Microscopy Simulation* (JEMS)^[49]^ software program with the use of the SCXRD data of **1**. The reader is referred to the Supporting Information document for further experimental details.

**Figure 4:**
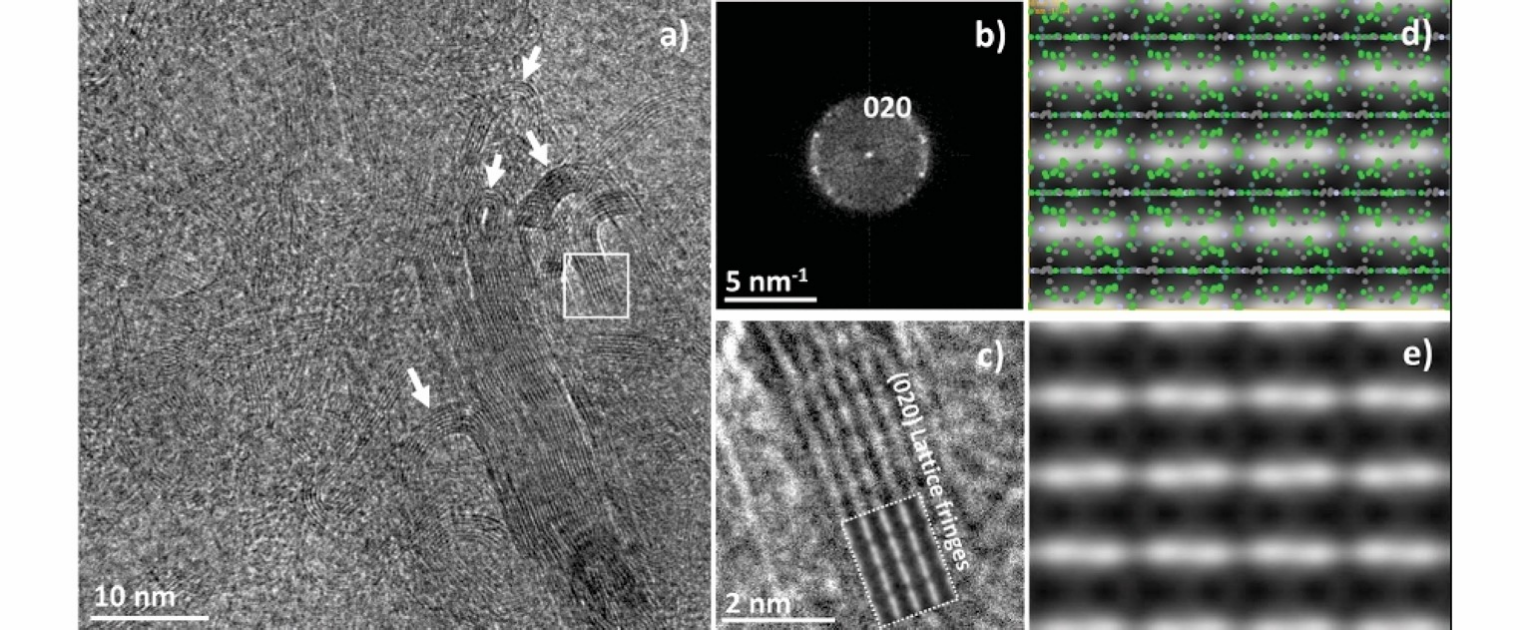
a) HRTEM image of **1** showing lattice fringes covering the entire imaged area of the material, b) diffractogram of the solid shown in panel (a), highlighting the diffracted intensity of **1** and indexed using simulated electron diffraction patterns, c) magnified view of the lattice fringes in the area marked in panel (a) with a white square, showing the experimental {020} lattice planes of **1** (viewed along the [$00\bar{1}$ ] direction); the dashed region encloses the relevant HRTEM simulation showing the excellent agreement with the experimental lattice fringes, d) an overlap of the simulated HRTEM image of **1** in the [001] zone axis with its atomic configuration projected along the [$00\bar{1}$ ] direction, e) the same simulated HRTEM image without the superimposed atomic coordinates of **1**.

The HRTEM analyses of a pristine powdered, mechanochemically prepared sample of **1** revealed that the specimen consists of micrometer‐sized particles with irregular morphologies (see Figure S20 in the Supporting Information document) typical for materials based on sp^2^ bonds, such as glassy carbon.^[50]^ Figure 4a shows a HRTEM image focused on a small area of such a particle. The observed lattice‐fringe contrast highlights the crystalline and layered nature of the material and its ability to locally bend without any evidence or signs of crystal fracture (the bent crystal regions are highlighted with arrows in Figure 4a). The experimental diffractogram in Figure 4b, collected on a particle area shown in Figure 4a, was successfully indexed using simulated electron diffraction patterns of **1**. The diffracted intensity was found to be distributed over arcs owing to the bending of the {020} crystal planes of **1**, which is consistent with the earlier reports of diffraction studies of plastically deformed molecular crystals.[^51^, ^52^] HRTEM simulations were also used to compare the HRTEM images of cocrystal **1** with the calculated ones (Figure 4c–e) to further validate the accuracy of the established crystal structure models.

We note that high mechanical flexibility and plastic behavior were previously observed in single‐ and multi‐component molecular crystals, and that the such flexibility and plasticity may be attributed to the presence of low frictional planes in their structure and the dynamic nature of self‐sorting intermolecular interactions (such as weak van der Waals interactions and halogen bonds).[^53^, ^54^, ^55^, ^56^] It is still unclear, however, whether the inherent conformational flexibility of **PEG‐DME** also plays a meaningful role in the apparent plastic deformation of **1**.

**Figure 5:**
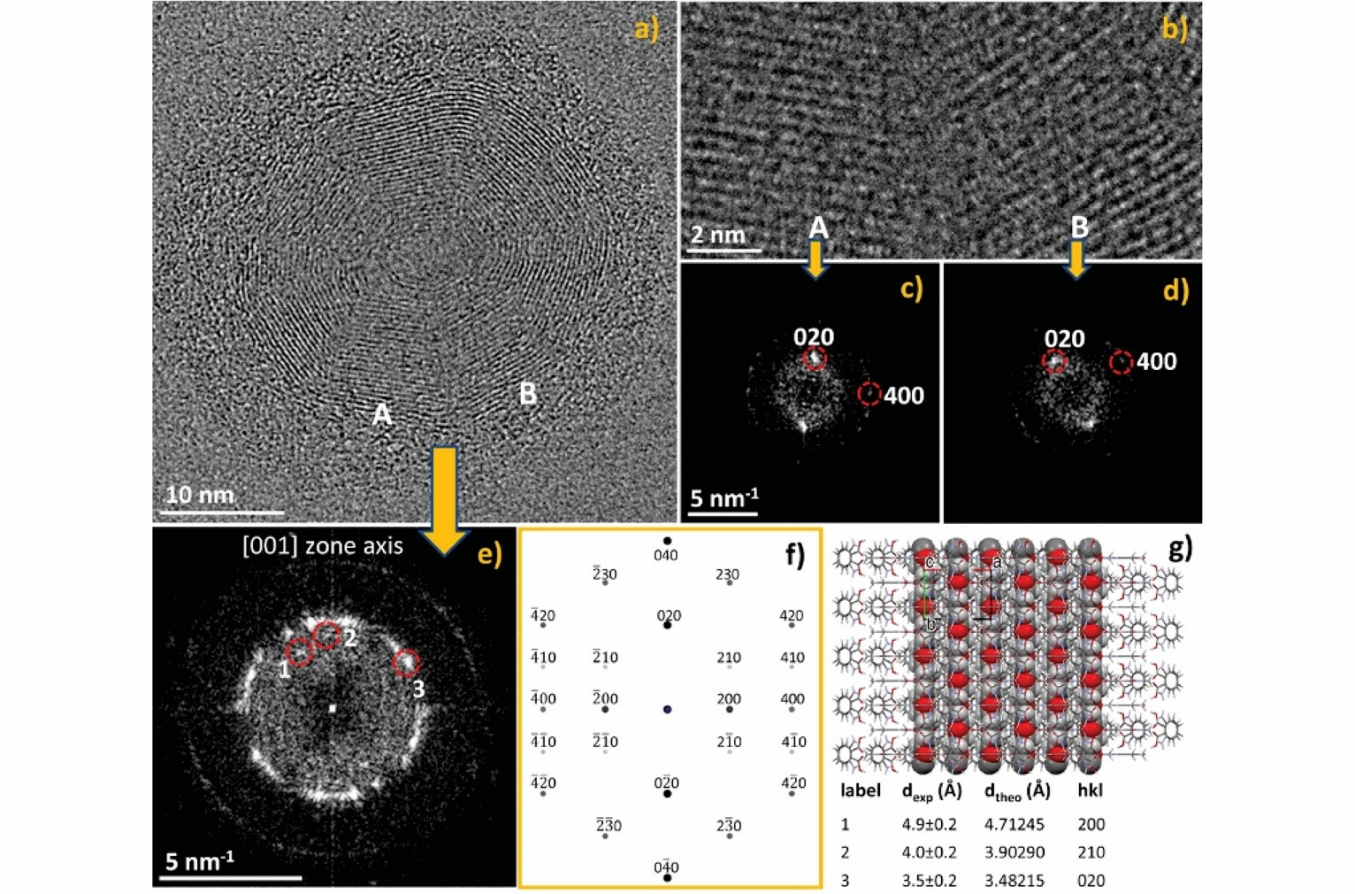
a) HRTEM image of a toroid particle of **1** with lattice fringes emerging from {020} lattice planes (viewed along the [001] zone axis); b) magnified view of the areas marked **A** and **B** in panel (a); c,d) diffractograms from areas **A** and **B** shown in panel (b), e) diffractogram from the entire toroid nanoparticle; f) calculated [001] zone axis electron diffraction pattern of **1**, g) the orientation of the crystal structure of **1** viewed along the crystallographic c‐axis direction and parallel to the direction of the electron beam. The table lists the measured (d_exp_) and calculated (d_theo_) lattice spacings for **1** (including Miller indices) used to index the diffractogram of the toroid crystal.

The high mechanical flexibility of **1**, and its durability under harsh milling conditions, is further underpinned by its ability to form a high number of toroidal nanoparticles with small inner radii and high curvatures (Figure 5), while maintaining its structural integrity. HRTEM imaging showed that continuity of the lattice fringes is preserved throughout the toroid (Figures 5a, see also Figures S21 and S22 in the Supporting Information document), although the peculiarities of the lattice fringes in the highly bent regions of the material suggest a high concentration of crystal structural defects (Figure 5b). Nevertheless, the obtained diffractograms and the relevant atomic resolution images show that these regions are indeed crystalline (Figure 5c–e). And while it is currently unclear whether the presumed defects in these areas are introduced by ball‐milling, or whether they are solely a consequence of the crystal deformation, we deem it possible that some of these defects could be also areas where the opposite ends of a straight single‐crystalline particle join to form the toroid. The ability of this material to flawlessly join the ends of a crystalline particle leads us to cautiously propose that polymer cocrystals should be considered as feasible model compounds for the development of crystalline materials with “self‐healing” properties.[^57^, ^58^, ^59^]

## Conclusion

We have demonstrated that thermal properties of polymers can be improved through cocrystallization without compromising their mechanical flexibility. Specifically, the cocrystallization of **PEG‐DME** with **caf** and **ana** resulted in a material with a melting point of 98 °C, as compared to a melting point of 36 °C for **PEG‐DME**. Crystal engineering design strategies were then used to identify **6Fana** as a suitable molecular building block for the formation of an isostructural cocrystal that has an appreciably higher melting point of 128 °C. The miscibility of the two cocrystals then enabled the fine‐tuning of the melting behavior of the **PEG‐DME** cocrystals in the 98–128 °C temperature range. We note that the ability to thermally stabilize polymers without compromising their mechanical flexibility has important implications for the development of pharmaceuticals, polymers for electronic applications and other materials with targeted properties. For example, we postulate that the use of polymers as cocrystal formers could enhance the tabletability of otherwise brittle pharmaceutical solids, while the use of small molecules as cocrystal formers could enhance the crystallinity and thermal stability of organic semiconducting polymers. We are currently investigating the mechanisms through which these multicomponent solids retain their mechanical flexibility. Further efforts are focused on probing the cocrystallization of other types of polymers to derive generally workable design guidelines^[60]^ for the discovery and development of polymer cocrystals.

## Conflict of interest

The authors declare no conflict of interest.

1

## Supporting information

As a service to our authors and readers, this journal provides supporting information supplied by the authors. Such materials are peer reviewed and may be re‐organized for online delivery, but are not copy‐edited or typeset. Technical support issues arising from supporting information (other than missing files) should be addressed to the authors.

Supporting Information

Supporting Information

Supporting Information

Supporting Information

Supporting Information

## Data Availability

The data that support the findings of this study are available in the supplementary material of this article.
