# Supplementary material for: Modulating Thermal Properties of Polymers through Crystal Engineering
Source: Angew Chem Int Ed Engl. 2023 Feb 1;62(19):e202212688. doi: 10.1002/anie.202212688 (PMC10947328; doi:10.1002/anie.202212688)

checkCIF/PLATON report

You have not supplied any structure factors. As a result the full set of tests cannot be run.

THIS REPORT IS FOR GUIDANCE ONLY. IF USED AS PART OF A REVIEW PROCEDURE FOR PUBLICATION, IT SHOULD NOT REPLACE THE EXPERTISE OF AN EXPERIENCED CRYSTALLOGRAPHIC REFEREE.

No syntax errors found. CIF dictionary Interpreting this report

Datablock: 1_xstr1501

```
Bond precision:   C-C = 0.0034 A          Wavelength=1.54184

Cell:             a=6.98249(19)     b=38.1732(8)       c=9.4519(2)
                  alpha=90          beta=90.206(2)     gamma=90
Temperature:      150 K
```

	Calculated	Reported
Volume	2519.33(10)	2519.33(11)
Space group	P 21/c	P 21/c
Hall group	-P 2ybc	-P 2ybc
Moiety formula	C8 H10 N4 O2, 2(C7 H7 N O2), C2 H4 O	C8 H10 N4 O2, 2(C7 H7 N O2), C2 H4 O
Sum formula	C24 H28 N6 O7	C24 H28 N6 O7
Mr	512.52	512.52
Dx,g cm-3	1.351	1.351
Z	4	4
Mu (mm-1)	0.848	0.848
F000	1080.0	1080.0
F000'	1083.64	
h,k,lmax	8,45,11	8,45,11
Nref	4449	4437
Tmin,Tmax	0.950,0.967	0.766,1.000
Tmin'	0.866	

```
Correction method= # Reported T Limits: Tmin=0.766 Tmax=1.000
AbsCorr = GAUSSIAN

Data completeness= 0.997          Theta(max)= 66.590

R(reflections)= 0.0532( 3758)                   wR2(reflections)= 0.1160( 4437)

S = 1.123                  Npar= 371
```

The following ALERTS were generated. Each ALERT has the format
test-name_ALERT_alert-type_alert-level.
Click on the hyperlinks for more details of the test.

Alert level C

```
PLAT250_ALERT_2_C Large U3/U1 Ratio for Average U(i,j) Tensor ....          2.1 Note
PLAT250_ALERT_2_C Large U3/U1 Ratio for Average U(i,j) Tensor ....          2.1 Note
```

Alert level G

```
PLAT002_ALERT_2_G Number of Distance or Angle Restraints on AtSite          20 Note
PLAT004_ALERT_5_G Polymeric Structure Found with Maximum Dimension           1 Info
PLAT007_ALERT_5_G Number of Unrefined Donor-H Atoms .............            9 Report
PLAT171_ALERT_4_G The CIF-Embedded .res File Contains EADP Records          10 Report
PLAT175_ALERT_4_G The CIF-Embedded .res File Contains SAME Records           1 Report
PLAT302_ALERT_4_G Anion/Solvent/Minor-Residue Disorder (Resd  3  )        100% Note
PLAT302_ALERT_4_G Anion/Solvent/Minor-Residue Disorder (Resd  4  )        100% Note
PLAT720_ALERT_4_G Number of Unusual/Non-Standard Labels .........            6 Note
PLAT811_ALERT_5_G No ADDSYM Analysis: Too Many Excluded Atoms ....           ! Info
PLAT860_ALERT_3_G Number of Least-Squares Restraints ............           23 Note
PLAT883_ALERT_1_G No Info/Value for _atom_sites_solution_primary .     Please Do !
PLAT933_ALERT_2_G Number of HKL-OMIT Records in Embedded .res File          11 Note
```

0 **ALERT level A** = Most likely a serious problem - resolve or explain
0 **ALERT level B** = A potentially serious problem, consider carefully
2 **ALERT level C** = Check. Ensure it is not caused by an omission or oversight
12 **ALERT level G** = General information/check it is not something unexpected

1 ALERT type 1 CIF construction/syntax error, inconsistent or missing data
4 ALERT type 2 Indicator that the structure model may be wrong or deficient
1 ALERT type 3 Indicator that the structure quality may be low
5 ALERT type 4 Improvement, methodology, query or suggestion
3 ALERT type 5 Informative message, check

It is advisable to attempt to resolve as many as possible of the alerts in all categories. Often the minor alerts point to easily fixed oversights, errors and omissions in your CIF or refinement strategy, so attention to these fine details can be worthwhile. In order to resolve some of the more serious problems it may be necessary to carry out additional measurements or structure refinements. However, the purpose of your study may justify the reported deviations and the more serious of these should normally be commented upon in the discussion or experimental section of a paper or in the "special_details" fields of the CIF. checkCIF was carefully designed to identify outliers and unusual parameters, but every test has its limitations and alerts that are not important in a particular case may appear. Conversely, the absence of alerts does not guarantee there are no aspects of the results needing attention. It is up to the individual to critically assess their own results and, if necessary, seek expert advice.

Publication of your CIF in IUCr journals

A basic structural check has been run on your CIF. These basic checks will be run on all CIFs submitted for publication in IUCr journals (*Acta Crystallographica*, *Journal of Applied Crystallography*, *Journal of Synchrotron Radiation*); however, if you intend to submit to *Acta Crystallographica Section C* or *E* or *IUCrData*, you should make sure that full publication checks are run on the final version of your CIF prior to submission.

Publication of your CIF in other journals

Please refer to the *Notes for Authors* of the relevant journal for any special instructions relating to CIF submission.

PLATON version of 18/05/2022; check.def file version of 17/05/2022

Datablock 1_xstr1501 - ellipsoid plot

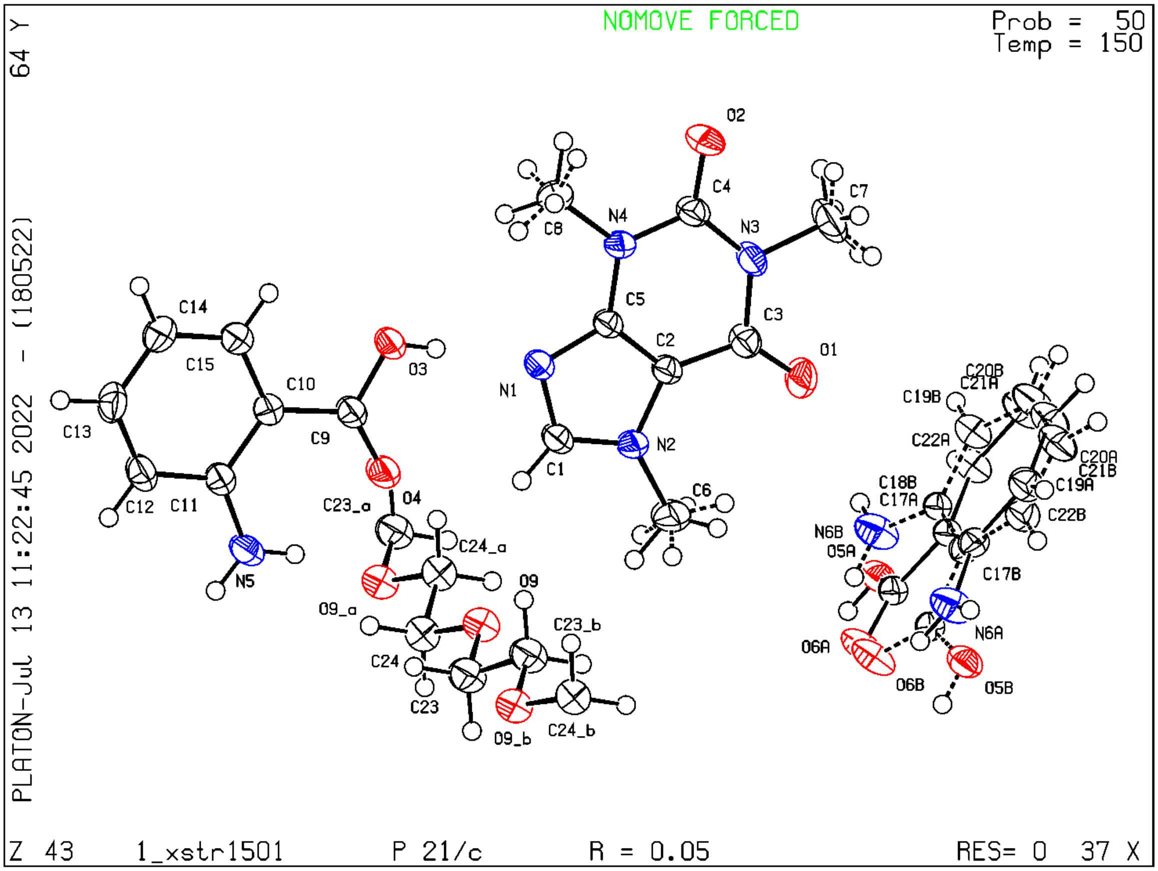

Supplement: Supplementary file 3 — Supporting Information [file ANIE-62-0-s001.pdf]
