# Supplementary material for: Modulating Thermal Properties of Polymers through Crystal Engineering
Source: Angew Chem Int Ed Engl. 2023 Feb 1;62(19):e202212688. doi: 10.1002/anie.202212688 (PMC10947328; doi:10.1002/anie.202212688)

checkCIF/PLATON report

You have not supplied any structure factors. As a result the full set of tests cannot be run.

THIS REPORT IS FOR GUIDANCE ONLY. IF USED AS PART OF A REVIEW PROCEDURE FOR PUBLICATION, IT SHOULD NOT REPLACE THE EXPERTISE OF AN EXPERIENCED CRYSTALLOGRAPHIC REFEREE.

No syntax errors found. CIF dictionary Interpreting this report

Datablock: 2_xstr1502

Bond precision: C-C = 0.0045 A Wavelength=1.54184

Cell:	a=9.46228(13)	b=38.3220(6)	c=6.96023(12)
	alpha=90	beta=90	gamma=90
Temperature:	150 K		

	Calculated	Reported
Volume	2523.87(7)	2523.87(7)
Space group	P n a 21	P n a 21
Hall group	P 2c -2n	P 2c -2n
Moiety formula	C8 H10 N4 O2, 2(C7 H6 F N O2), C2 H4 O	C8 H10 N4 O2, 2(C7 H6 F N O2), C2 H4 O
Sum formula	C24 H26 F2 N6 O7	C24 H26 F2 N6 O7
Mr	548.51	548.51
Dx,g cm-3	1.444	1.444
Z	4	4
Mu (mm-1)	1.004	1.004
F000	1144.0	1144.0
F000'	1148.21	
h,k,lmax	11,48,8	11,48,8
Nref	5360[2907]	5038
Tmin,Tmax	0.897,0.980	0.885,0.987
Tmin'	0.818	

Correction method= # Reported T Limits: Tmin=0.885 Tmax=0.987
AbsCorr = ANALYTICAL

Data completeness= 1.73/0.94 Theta(max)= 77.623

R(reflections)= 0.0440(4714) wR2(reflections)= 0.1138(5038)

S = 1.048 Npar= 392

The following ALERTS were generated. Each ALERT has the format
test-name_ALERT_alert-type_alert-level.
Click on the hyperlinks for more details of the test.

Alert level C

```
PLAT250_ALERT_2_C Large U3/U1 Ratio for Average U(i,j) Tensor ....        2.1 Note
PLAT250_ALERT_2_C Large U3/U1 Ratio for Average U(i,j) Tensor ....        2.3 Note
PLAT340_ALERT_3_C Low Bond Precision on  C-C Bonds ..............     0.0045 Ang.
```

Alert level G

```
PLAT002_ALERT_2_G Number of Distance or Angle Restraints on AtSite         22 Note
PLAT004_ALERT_5_G Polymeric Structure Found with Maximum Dimension          1 Info
PLAT007_ALERT_5_G Number of Unrefined Donor-H Atoms .............           9 Report
PLAT012_ALERT_1_G N.O.K.   _shelx_res_checksum Found in CIF ......     Please Check
PLAT171_ALERT_4_G The CIF-Embedded .res File Contains EADP Records         11 Report
PLAT175_ALERT_4_G The CIF-Embedded .res File Contains SAME Records          1 Report
PLAT302_ALERT_4_G Anion/Solvent/Minor-Residue Disorder (Resd  3  )       100% Note
PLAT302_ALERT_4_G Anion/Solvent/Minor-Residue Disorder (Resd  4  )       100% Note
PLAT720_ALERT_4_G Number of Unusual/Non-Standard Labels .........           6 Note
PLAT811_ALERT_5_G No ADDSYM Analysis: Too Many Excluded Atoms ....          ! Info
PLAT860_ALERT_3_G Number of Least-Squares Restraints ............          27 Note
PLAT883_ALERT_1_G No Info/Value for _atom_sites_solution_primary .     Please Do !
PLAT933_ALERT_2_G Number of HKL-OMIT Records in Embedded .res File         11 Note
PLAT965_ALERT_2_G The SHELXL WEIGHT Optimisation has not Converged     Please Check
```

0 **ALERT level A** = Most likely a serious problem - resolve or explain
0 **ALERT level B** = A potentially serious problem, consider carefully
3 **ALERT level C** = Check. Ensure it is not caused by an omission or oversight
14 **ALERT level G** = General information/check it is not something unexpected

2 ALERT type 1 CIF construction/syntax error, inconsistent or missing data
5 ALERT type 2 Indicator that the structure model may be wrong or deficient
2 ALERT type 3 Indicator that the structure quality may be low
5 ALERT type 4 Improvement, methodology, query or suggestion
3 ALERT type 5 Informative message, check

It is advisable to attempt to resolve as many as possible of the alerts in all categories. Often the minor alerts point to easily fixed oversights, errors and omissions in your CIF or refinement strategy, so attention to these fine details can be worthwhile. In order to resolve some of the more serious problems it may be necessary to carry out additional measurements or structure refinements. However, the purpose of your study may justify the reported deviations and the more serious of these should normally be commented upon in the discussion or experimental section of a paper or in the "special_details" fields of the CIF. checkCIF was carefully designed to identify outliers and unusual parameters, but every test has its limitations and alerts that are not important in a particular case may appear. Conversely, the absence of alerts does not guarantee there are no aspects of the results needing attention. It is up to the individual to critically assess their own results and, if necessary, seek expert advice.

Publication of your CIF in IUCr journals

A basic structural check has been run on your CIF. These basic checks will be run on all CIFs submitted for publication in IUCr journals (*Acta Crystallographica*, *Journal of Applied Crystallography*, *Journal of Synchrotron Radiation*); however, if you intend to submit to *Acta Crystallographica Section C* or *E* or *IUCrData*, you should make sure that full publication checks are run on the final version of your CIF prior to submission.

Publication of your CIF in other journals

Please refer to the *Notes for Authors* of the relevant journal for any special instructions relating to CIF submission.

PLATON version of 18/05/2022; check.def file version of 17/05/2022

Datablock 2_xstr1502 - ellipsoid plot

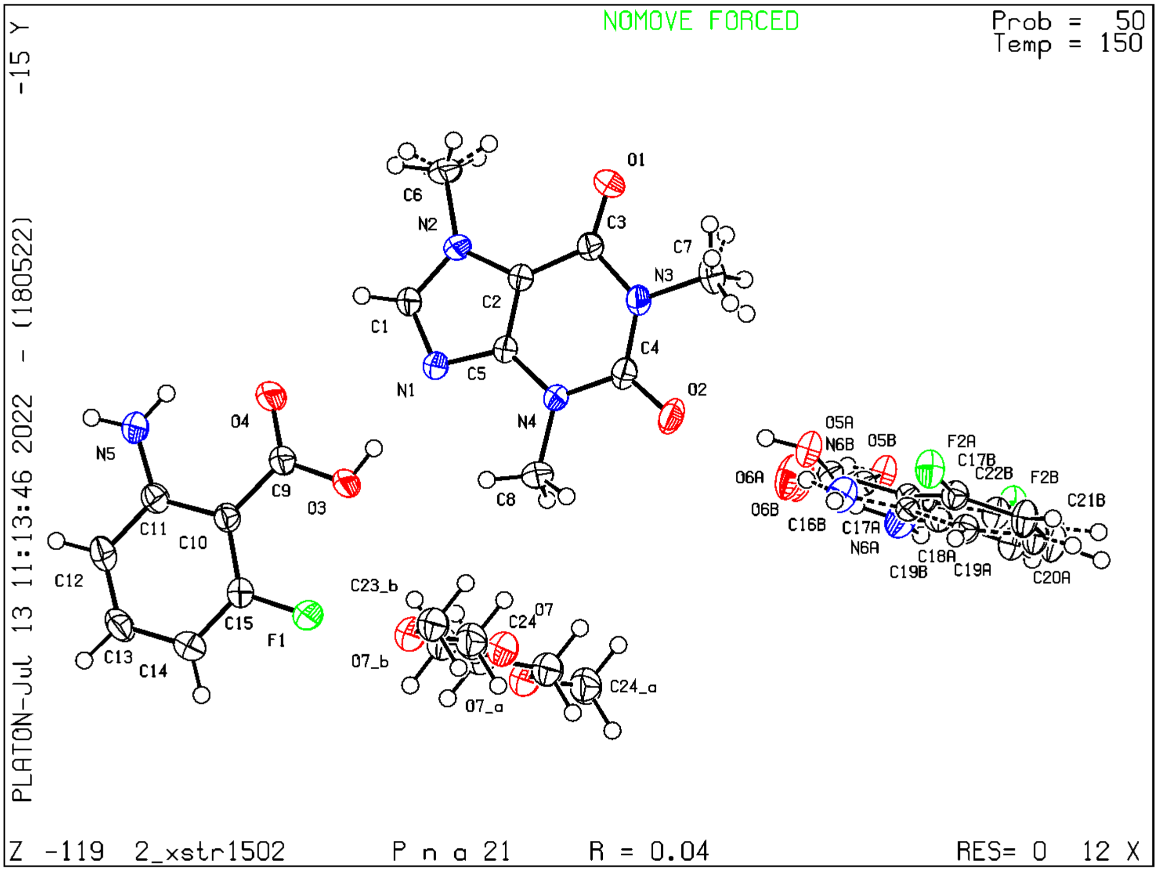

Supplement: Supplementary file 4 — Supporting Information [file ANIE-62-0-s005.pdf]
