# Supplementary material for: Modulating Thermal Properties of Polymers through Crystal Engineering
Source: Angew Chem Int Ed Engl. 2023 Feb 1;62(19):e202212688. doi: 10.1002/anie.202212688 (PMC10947328; doi:10.1002/anie.202212688)
Supplement: Supplementary file 5 — Supporting Information [file ANIE-62-0-s004.pdf]

Angewandte Chemie
Eine Zeitschrift der Gesellschaft Deutscher Chemiker

Supporting Information

Modulating Thermal Properties of Polymers through Crystal Engineering

L. S. Germann, E. Carlino, A. Taurino, O. V. Magdysyuk, D. Voinovich, R. E. Dinnebier, D.-K. Bučar, D. Hasa**

Supporting Information
©Wiley-VCH 2021
69451 Weinheim, Germany

Modulating thermal properties of polymers through crystal engineering

Luzia S. Germann, Elvio Carlino, Antonietta Taurino, Oxana V. Magdysyuk, Dario Voinovich, Robert E. Dinnebier, Dejan-Krešimir Bučar* and Dritan Hasa*

Table of Contents

1. Materials 2

2. Discovery of polymer cocrystals *via* in situ monitoring of milling experiments 2

3. Mechanochemical synthesis of polymer cocrystals and their solid solutions 2

4. Powder X-ray diffraction and thermal analyses of polymer cocrystals and their solid solutions 3

5. Preparation of single crystals of **1** and **2** 13

6. Single crystal X-ray diffraction studies 13

7. High resolution TEM analyses 14

9. Solid-form informatics 15

10. References 16

1. Materials

Anhydrous caffeine (**caf**) (99%), anthranilic acid (**ana**) (99%), 6-fluoroanthranilic acid (**6F-ana**) (98%), poly(ethylene glycol) 1000 (**PEG-1000**) (M_n=1000 g mol^{-1}), poly(ethylene glycol) 3000 (**PEG-3000**) (M_n=3000 g mol^{-1}), poly(ethylene glycol) dimethyl ether 1000 (**PEG-DME**) (M_n=1050 g mol^{-1}) and all used solvents were purchased from *Sigma Aldrich*. All reagents were used as received.

2. Discovery of polymer cocrystals *via in situ* monitoring of milling experiments

The polymer cocrystals were discovered during *in situ* monitoring experiments of mechanochemical cocrystallisation reactions that have been previously studied in our groups and by others.[1-2] **Caf** and **ana** were milled in a 1:1 stoichiometic ratio (m_{total} = 400 mg) in the presence of 5 mg, 10 mg and 70 mg of **PEG-3000**. The milling assembly consisted of a X-ray transparent poly(methyl methacrylate) milling jar and two 7 mm steel balls (m = 1.38 g). The samples were prepared using a modified *Retsch MM400* shaker mill. The time-resolved powder X-ray diffraction (PXRD) monitoring of the cocrystallisation reactions was performed at the Powder Diffraction and Total Scattering Beamline P02.1 at the *Deutsches-Elektronen Synchrotron* (DESY) in Hamburg (Germany). The beamline operated at a fixed energy of *ca.* 60 keV (λ = 0.20709 Å).

The *in situ* monitoring showed that milling equivalent amounts of **caf** with **ana** in the presence of small amounts of **PEG-3000** (m = 5 mg and 10 mg) leads to the formation of the known cocrystal (**caf**)·(**ana**) – Form 1 (CSD reference code: ZOBCOK), while larger amounts of **PEG-3000** (70 mg of polymer for 400 mg of (**caf**)·(**ana**) mixture) lead to the formation of a ternary cocrystal, which was later shown to be isomorphous with (**PEG-DME**)·(**caf**)$_{23}$·(**ana**)$_{46}$ (**1**) (see Figure S1).

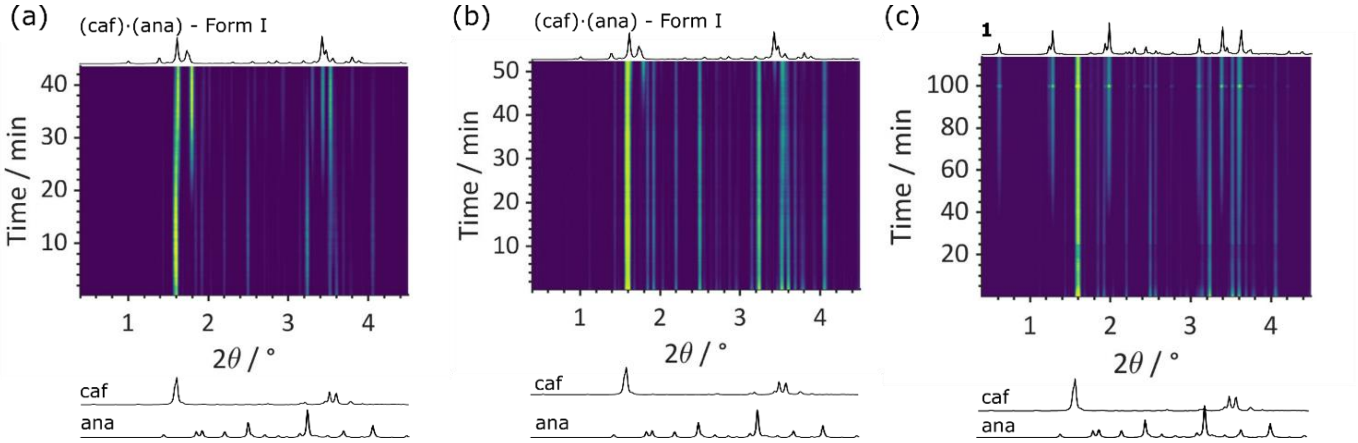

Figure S1. 2D PXRD plot of the *in situ* monitoring of the mechanochemical cocrystallization of **caf**, **ana** and **PEG-3000** with (a) 5 mg **PEG-3000** at a milling frequency of 30 Hz, (b) 10 mg **PEGDME-3000** at a milling frequency of 25 Hz, and (**c**) 70 mg **PEG-3000** at a milling frequency of 30 Hz. Calculated PXRD patterns (λ = 0.20709 Å) of the reactants, (**caf**)·(**ana**) Form I and cocrystal **1** are shown below and above the 2D plots, respectively.

3. Mechanochemical synthesis of polymer cocrystals and their solid solutions

The discovery of the **PEG-3000** cocrystal prompted the cocrystallisation of **PEG-DME** with **caf** and **ana** (and later also with **caf** and **6Fana**). Cocrystals (**PEG-DME**)·(**caf**)$_{23}$·(**ana**)$_{46}$ (**1**) and (**PEG-DME**)·(**caf**)$_{23}$·(**6Fana**)$_{46}$ (**2**), were prepared mechanochemically in the absence of any liquid additives using a *Retsch MM200* mixer mill. In a typical experiment, a physical mixture (m = 250 mg) of **caf**, **ana** (or **6F-ana**) and **PEG-DME** was added to a 15 mL screw stainless steel milling jar with two 7 mm steel milling balls, and subsequently milled for 60 min at 25 Hz.

Solid solutions composed of **1** and **2** in various ratios were also prepared mechanochemically and in the absence of any solvents. Specifically, about 250 mg of a physical mixture containing **caf**, **PEG-DME**, **ana** and **6Fana** was added to a 15 mL screw stainless steel milling jar and processed for 60 min at 25 Hz using two 7 mm steel milling balls. Two batches were prepared for each composition. All obtained solids were characterized both by powder X-ray diffraction and differential scanning calorimetry. The diffraction patterns and thermograms of all solids are shown in Section 4 of this document.

4. Powder X-ray diffraction and thermal analyses of polymer cocrystals and their solid solutions

Room temperature PXRD data sets were obtained using a *STOE Stadi-P* X-ray diffractometer equipped with a Cu tube set to 50 kV and 30 mA ($K\alpha1$ with λ = 1.5406 Å), a primary beam monochromator and *Dectris Mythen* detector. Measurements were made in thin-foil transmission mode with a 0.5° detector step in the 2θ range of 2° to 60°, and a counting time of 10 s per step. The data was acquired and treated using the *STOE WinX^{POW}* program (version 3.20). The diffractograms of the mechanochemically prepared polymer cocrystals and their solid solutions are shown in the following subsections of this text.

Rietveld refinements[3,4] of the solid solutions were performed using the *Topas* 4.2 program.[5] Since the monoclinic distortion of the low-symmetry structure of cocrystal **1** is very small, both crystal structures of **1** and **2** were considered for Rietveld refinements. The R_{wp} factor obtained from the Rietveld refinement was then used to determine in which space group the solid solution crystallized. Specifically, an orthorhombic setting was chosen in all cases where the Rietveld refinement resulted in an R_{wp} factor lower or similar to the R_{wp} factor being derived from a refinement based on the monoclinic structure. The unit cell parameters of the solid solutions are summarized in Table S1.

Table S1. Unit cell parameters of the solid solutions containing **PEG-DME**, **caf**, **ana** and **6Fana**, as determined by Rietveld analyses.

**composition** $\left[\frac{n(\boldsymbol{6Fana})}{n(\boldsymbol{ana})+n(\boldsymbol{6Fana})}\cdot 100\%\right]$	**space group**	***a* / Å**	***b* / Å**	***c* / Å**	***α* / °**	***β* / °**	***γ* / °**	***V* / Å³**
0 (cocrystal **1**)	$P2_1/c$	7.004610	38.39449	9.537508	90	90.26598	90	2564.97
12.5	$P2_1/c$	7.004808	38.39656	9.542547	90	90.27358	90	2566.54
25	$P2_1/c$	7.000267	38.37443	9.538878	90	90.25538	90	2562.42
37.5	$P2_1/c$	7.002340	38.39609	9.547475	90	90.28024	90	2566.97
50	$P2_1/c$	7.000113	38.39846	9.548562	90	90.27866	90	2566.56
62.5	$Pna2_1$	9.554016	38.42652	7.002823	90	90	90	2570.93
75	$Pna2_1$	9.554953	38.42492	7.002638	90	90	90	2571.01
87.5	$Pna2_1$	9.557571	38.43376	7.003617	90	90	90	2572.66
100 (cocrystal **2**)	$Pna2_1$	9.559556	38.43473	7.004054	90	90	90	2573.42

Differential scanning calorimetric (DSC) measurements were performed on a *Mettler Toledo DSC 3* instrument. Approximately 3 mg of each solid was weighed in a 40 µL alumina pan and covered with an alumina lid. Each sample was heated from 30 °C to 260 °C, with a heating rate of 10 °C min^{-1}. The samples were heated under the flow of dry nitrogen gas. The DSC curves were processed using the *Mettler STAR^{e}* data evaluation software (version 16.40). The thermograms of the polymer cocrystals and their solid solutions are shown in the following subsections of this text. The melting points of the studied solid solutions are shown in Table S2.

Table S2. Summary of the melting points of cocrystals **1** and **2**, and different solid solutions containing **PEG-DME**, **caf**, **ana** and **6Fana**. All cocrystals and solid solutions were prepared mechanochemically.

**composition**	**t_m(peak) / °C**	
$\left[\frac{n(\boldsymbol{6Fana})}{n(\boldsymbol{ana})+n(\boldsymbol{6Fana})}\cdot 100\%\right]$	**batch 1**	**batch 1**
0 (cocrystal **1**)	97.49	97.95
12.5	105.09	104.59
25	107.91	108.12
37.5	113.73	114.36
50	117.07	117.68
62.5	120.62	120.82
75	123.20	123.47
87.5	125.25	126.01
100 (cocrystal **2**)	127.43	128.61

4.1 Cocrystal (**PEG-DME**)·(**caf**)$_{23}$·(**ana**)$_{46}$ (**1**)

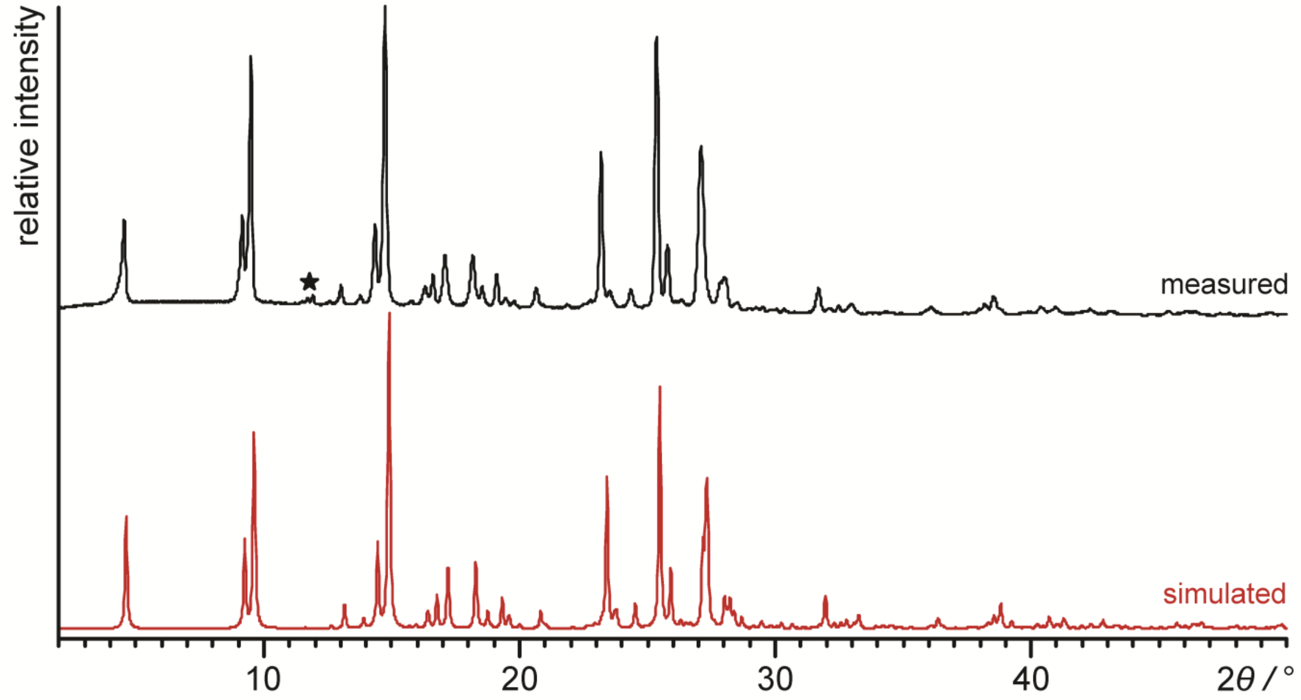

Figure S1. Measured diffractogram of the mechanochemically prepared (**PEG-DME**)·(**caf**)$_{23}$·(**ana**)$_{46}$ cocrystal (shown in black), as compared to its simulated diffractogram (derived from single crystal X-ray diffraction data, shown in red). The diffraction peaks at approximately 12° 2*θ* (highlighted using the ★ symbol) correspond to **caf** impurities (CSD reference code: NIWFEE06).

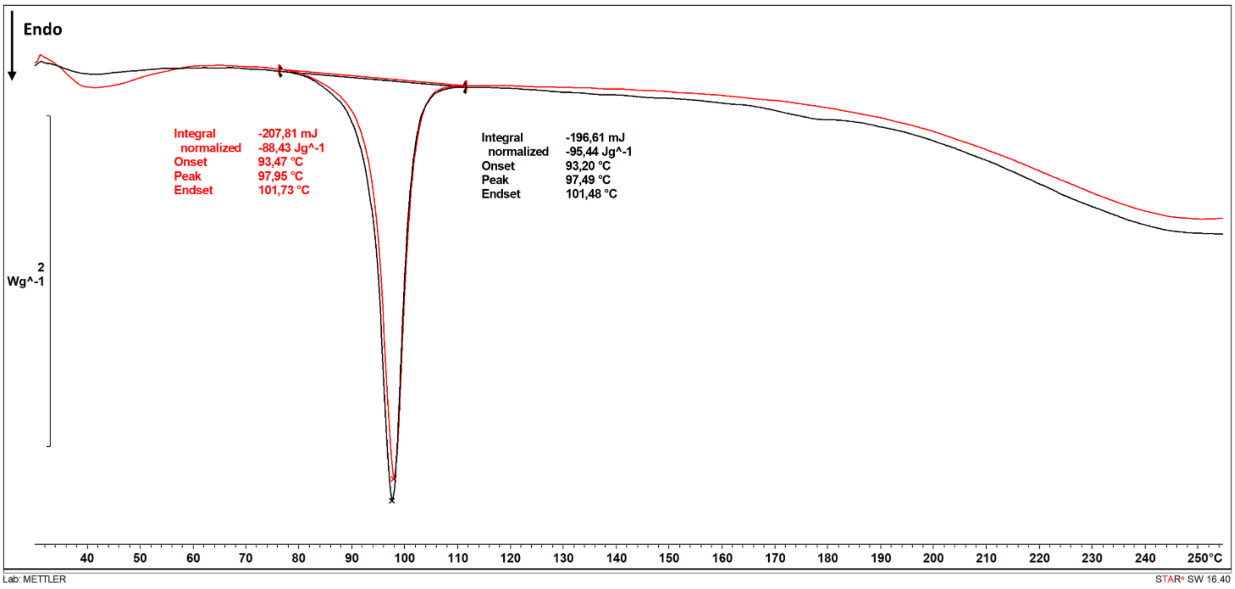

Figure S3. DSC thermograms of two different batches of cocrystal **1**.

4.2 Cocrystal (**PEG-DME**)·(**caf**)$_{23}$·(**6Fana**)$_{46}$ (**2**)

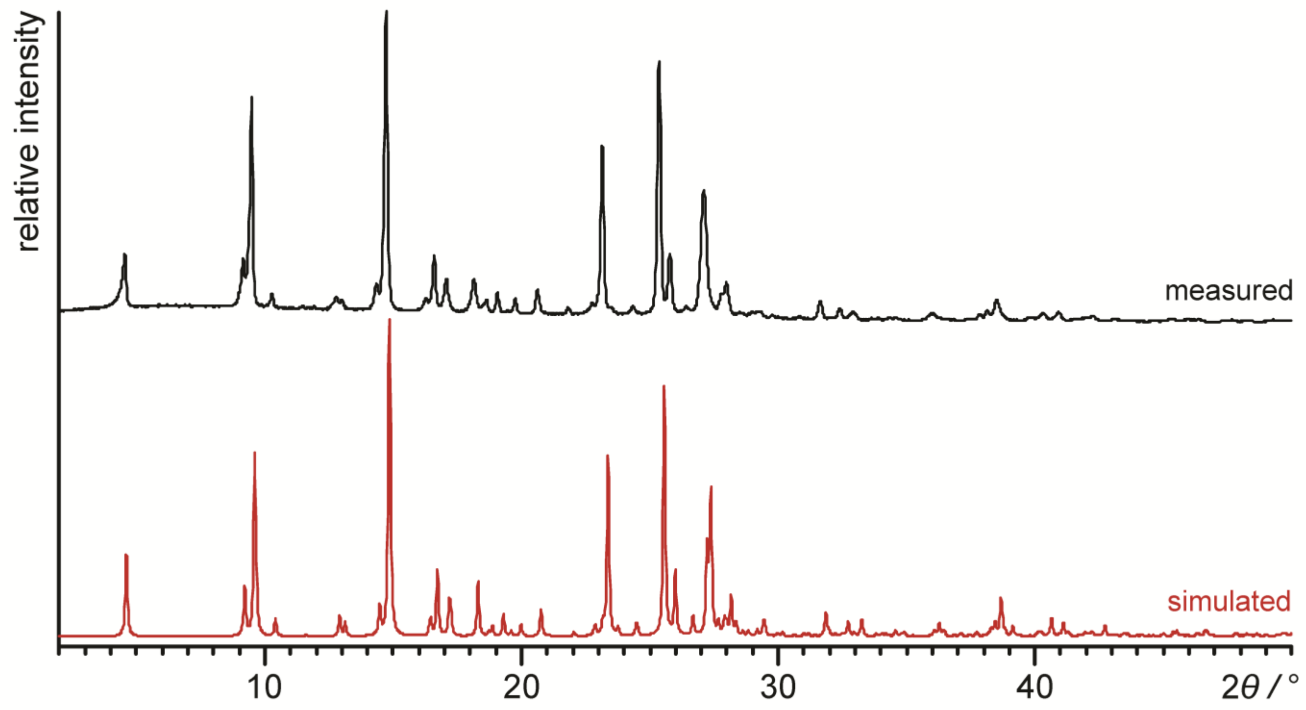

Figure S4. Measured diffractogram of the mechanochemically prepared **(PEG-DME)·(caf)$_{23}$·(6Fana)$_{46}$** cocrystal (shown in black), as compared to its simulated diffractogram (derived from single crystal X-ray diffraction data, shown in red).

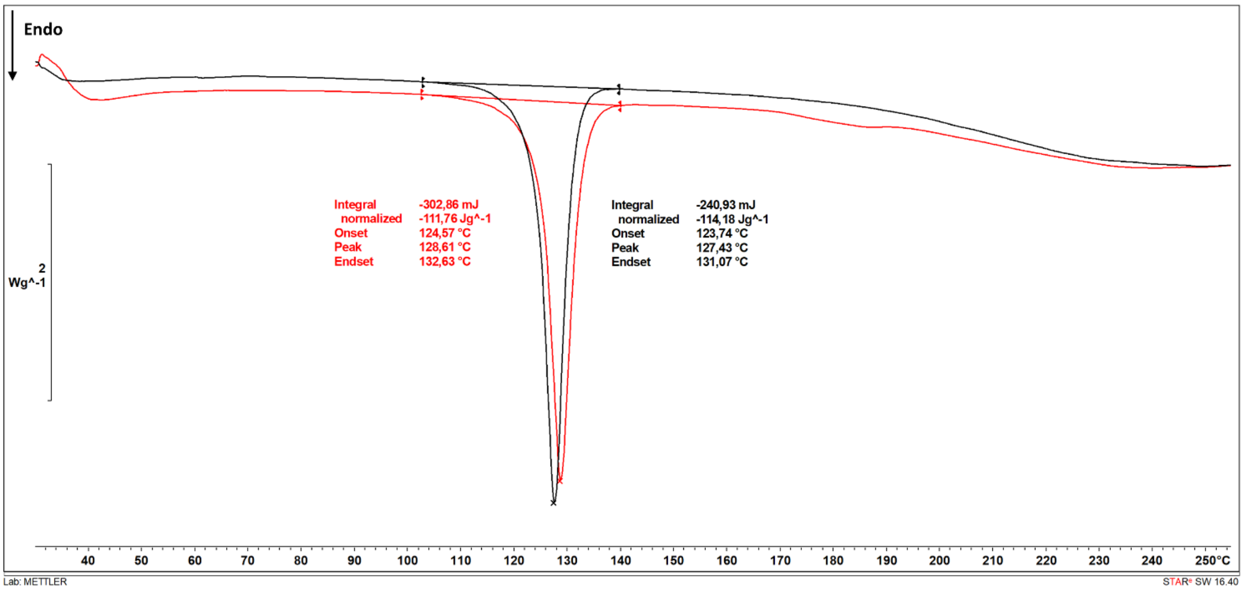

Figure S5. DSC thermograms of two different batches of cocrystal **2**.

*4.3 Solid solution (**PEG-DME**)·(**caf**)$_{23}$·(**ana**)$_{40.25}$·(**6Fana**)$_{5.75}$*

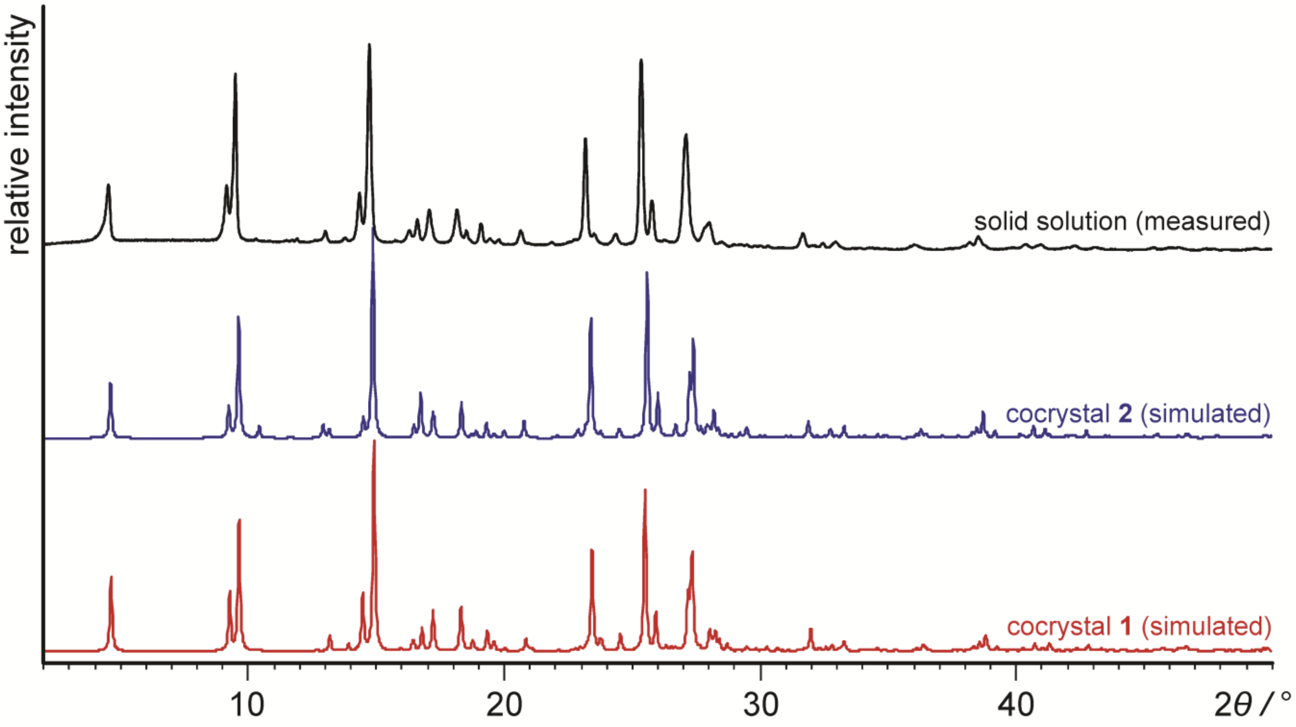

Figure S6. Measured diffractogram of the mechanochemically prepared (**PEG-DME**)·(**caf**)$_{23}$·(**ana**)$_{40.25}$·(**6Fana**)$_{5.75}$ cocrystal (shown in black), as compared to the simulated diffractogram of cocrystal **1** (derived from single crystal X-ray diffraction data, shown in red).

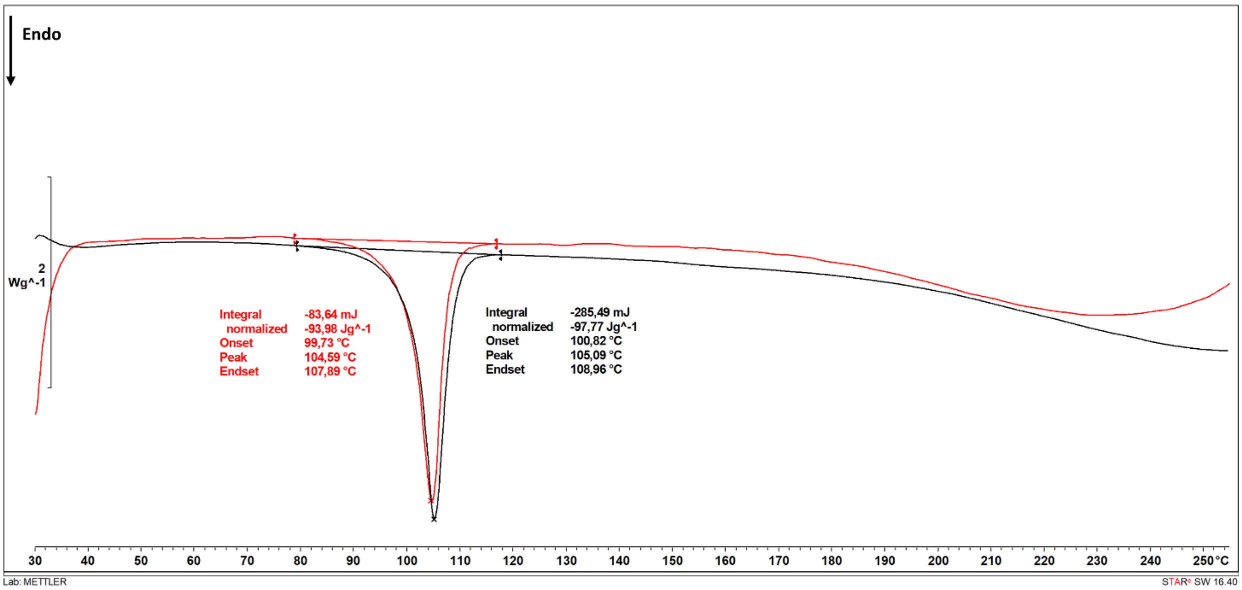

Figure S7. DSC thermograms of two different batches of the (**PEG-DME**)·(**caf**)$_{23}$·(**ana**)$_{40.25}$·(**6Fana**)$_{5.75}$ solid solution.

*4.4 Solid solution (**PEG-DME**)·(**caf**)$_{23}$·(**ana**)$_{34.5}$·(**6Fana**)$_{11.5}$*

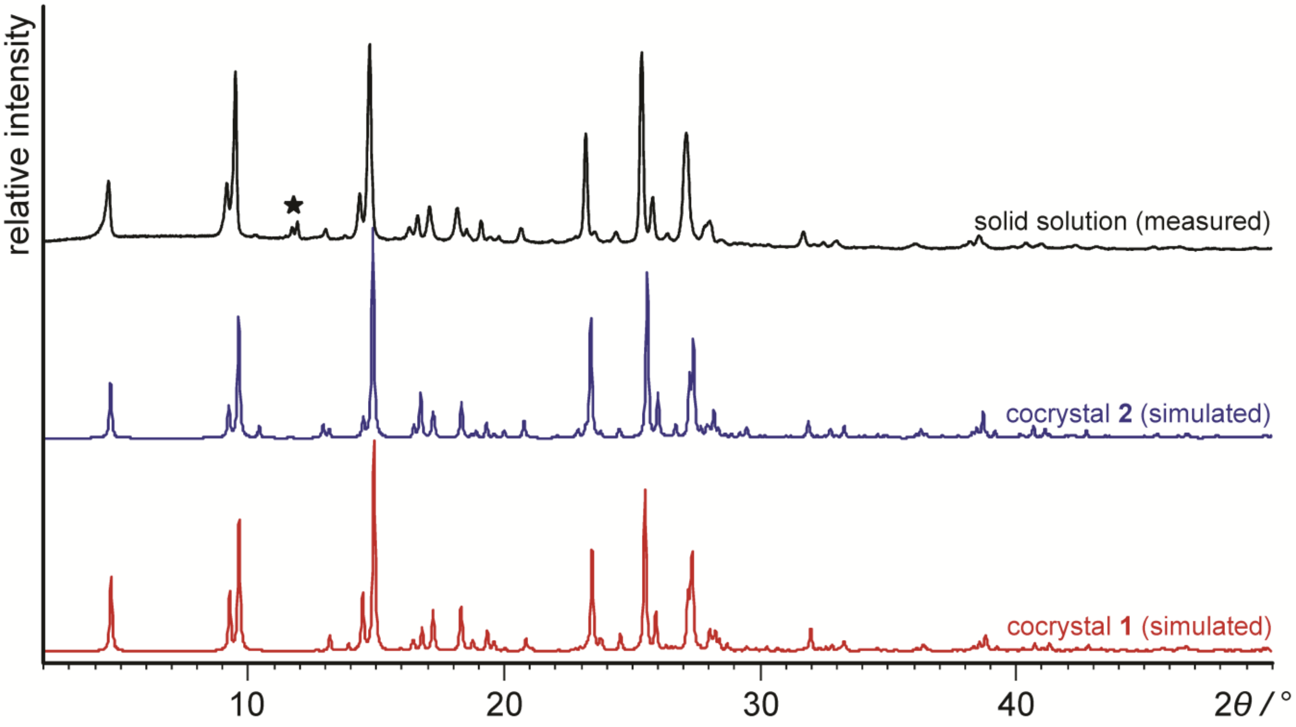

Figure S8. Measured diffractogram of the mechanochemically prepared (**PEG-DME**)·(**caf**)$_{23}$·(**ana**)$_{34.5}$·(**6Fana**)$_{11.5}$ cocrystal (shown in black), as compared to the simulated diffractogram of cocrystal **1** (derived from single crystal X-ray diffraction data, shown in red). The diffraction peaks at approximately 12° 2θ (highlighted using the ★ symbol) correspond to **caf** impurities (CSD reference code: NIWFEE06).

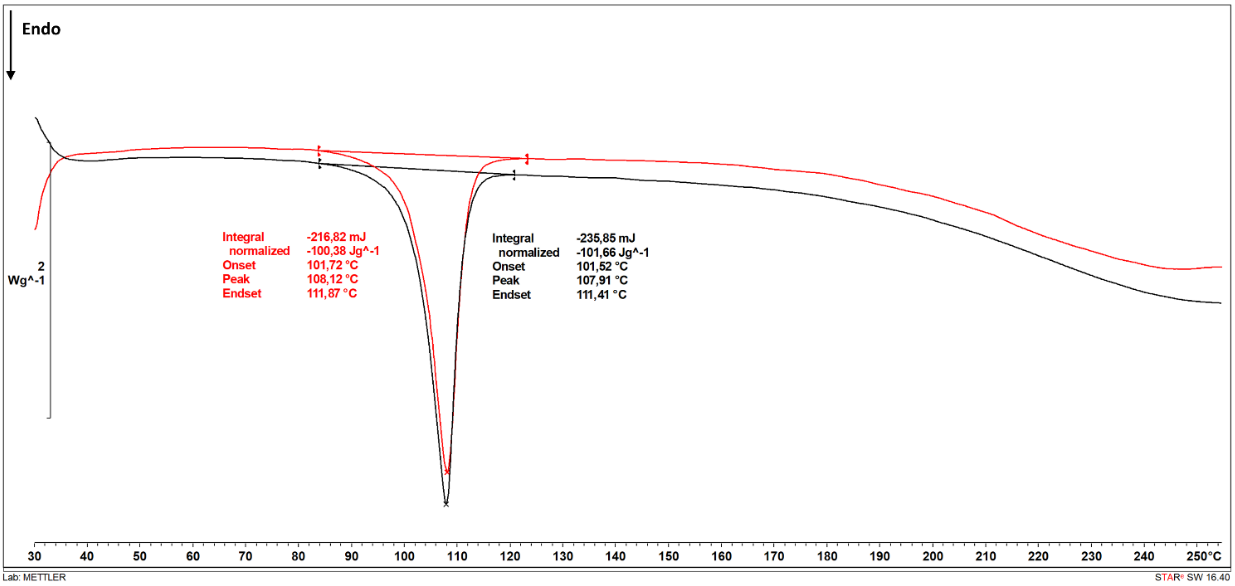

Figure S9. DSC thermograms of two different batches of the (**PEG-DME**)·(**caf**)$_{23}$·(**ana**)$_{34.5}$·(**6Fana**)$_{11.5}$ solid solution.

4.5 Solid solution (**PEG-DME**)·(**caf**)$_{23}$·(**ana**)$_{28.75}$·(**6Fana**)$_{17.25}$

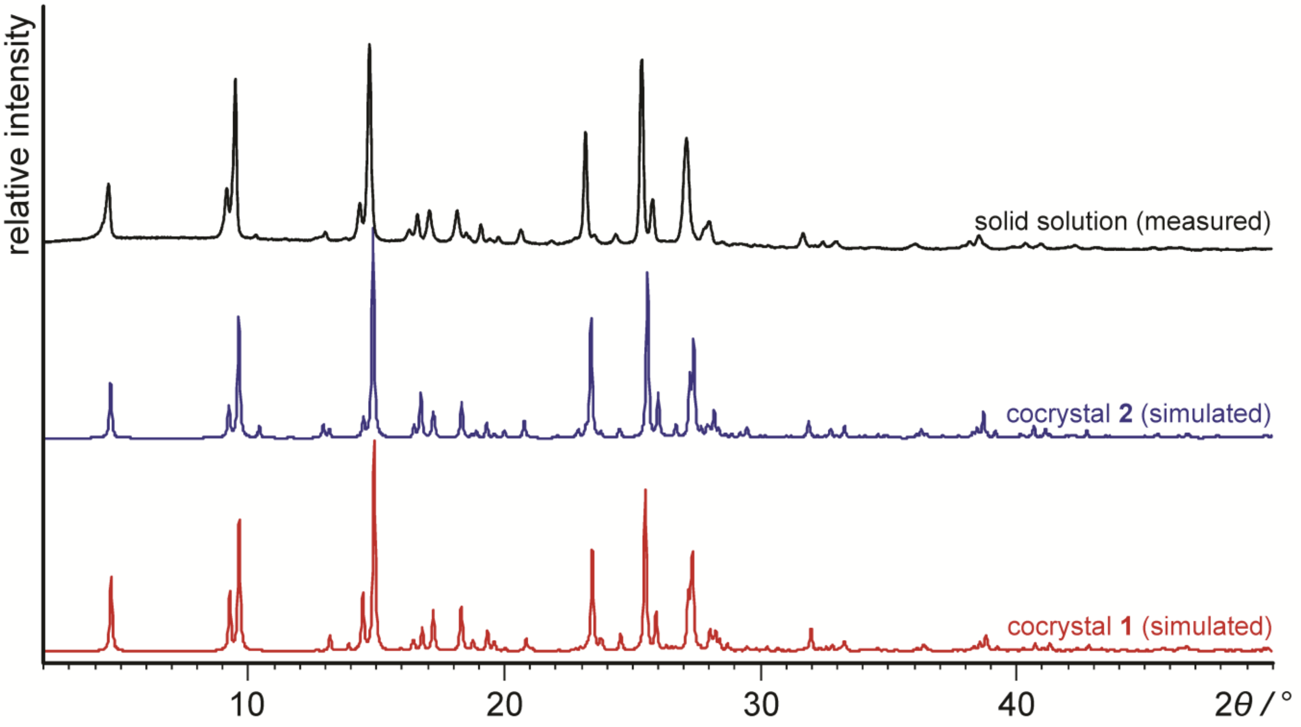

Figure S10. Measured diffractogram of the mechanochemically prepared (**PEG-DME**)·(**caf**)$_{23}$·(**ana**)$_{28.75}$·(**6Fana**)$_{17.25}$ cocrystal (shown in black), as compared to the calculated diffractogram of cocrystal **1** (derived from single crystal X-ray diffraction data, shown in red).

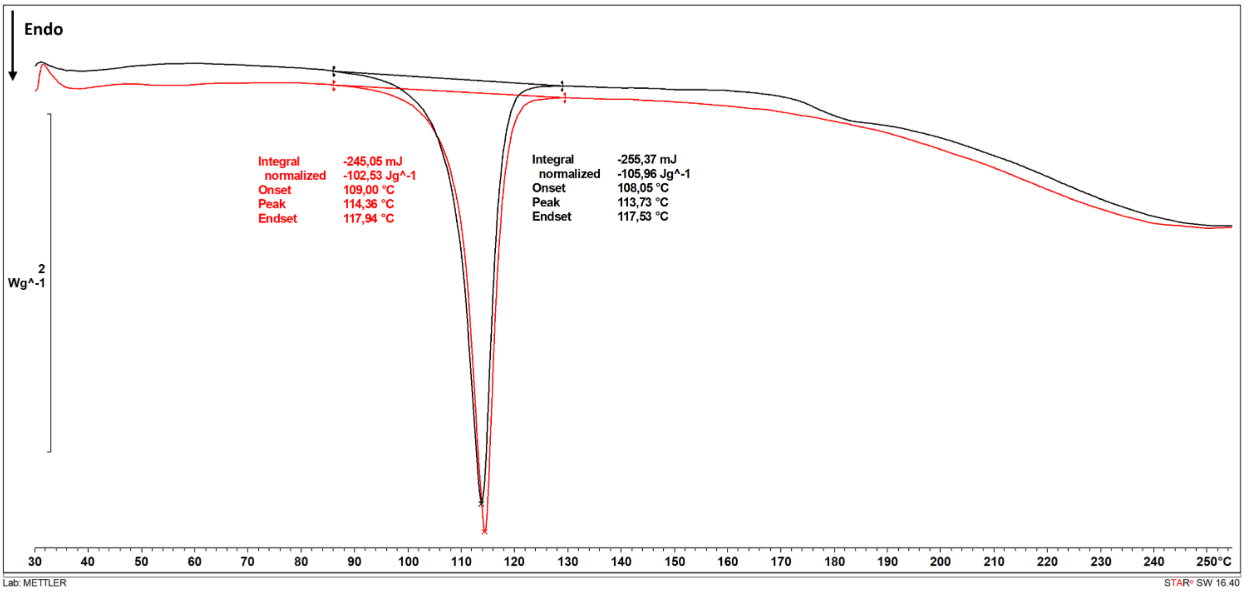

Figure S11. DSC thermograms of two different batches of the (**PEG-DME**)·(**caf**)$_{23}$·(**ana**)$_{28.75}$·(**6Fana**)$_{17.25}$ solid solution.

*4.6 Solid solution (**PEG-DME**)·(**caf**)$_{23}$·(**ana**)$_{23}$·(**6Fana**)$_{23}$*

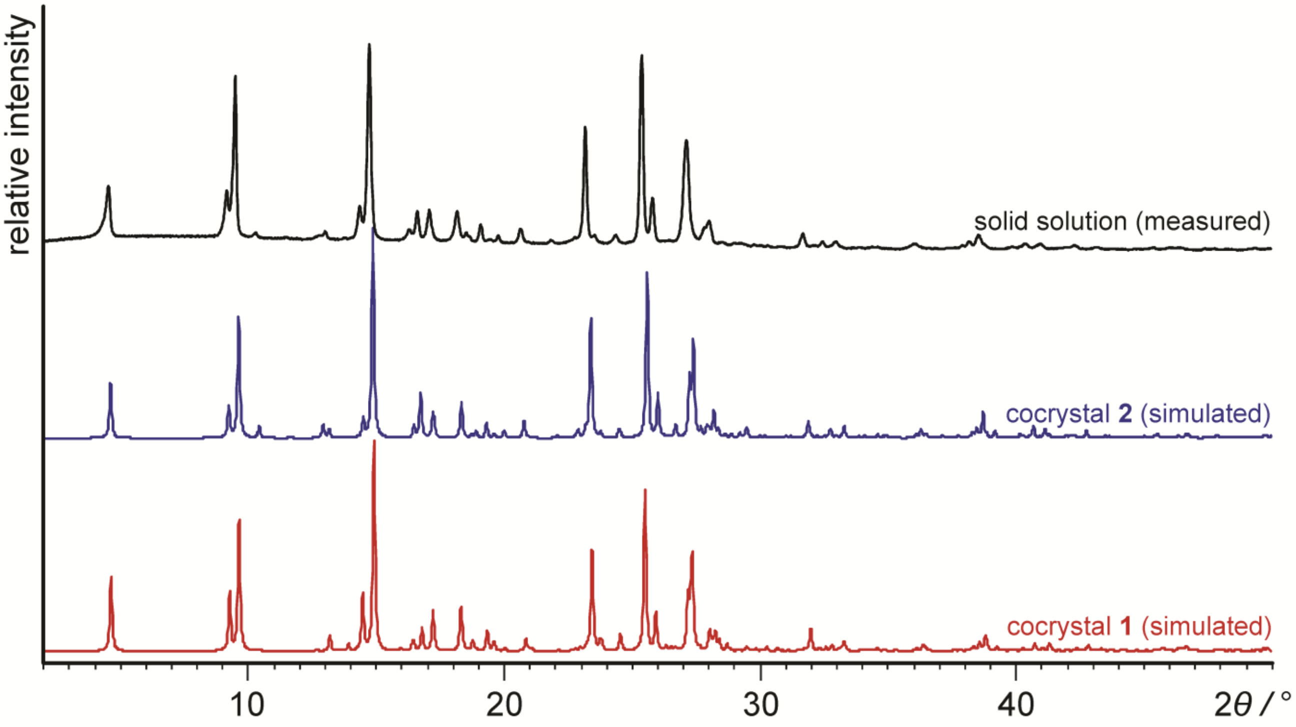

Figure S12. Measured diffractogram of the mechanochemically prepared (**PEG-DME**)·(**caf**)$_{23}$·(**ana**)$_{23}$·(**6Fana**)$_{23}$ cocrystal (shown in black), as compared to the calculated diffractogram of cocrystal **1** (derived from single crystal X-ray diffraction data, shown in red).

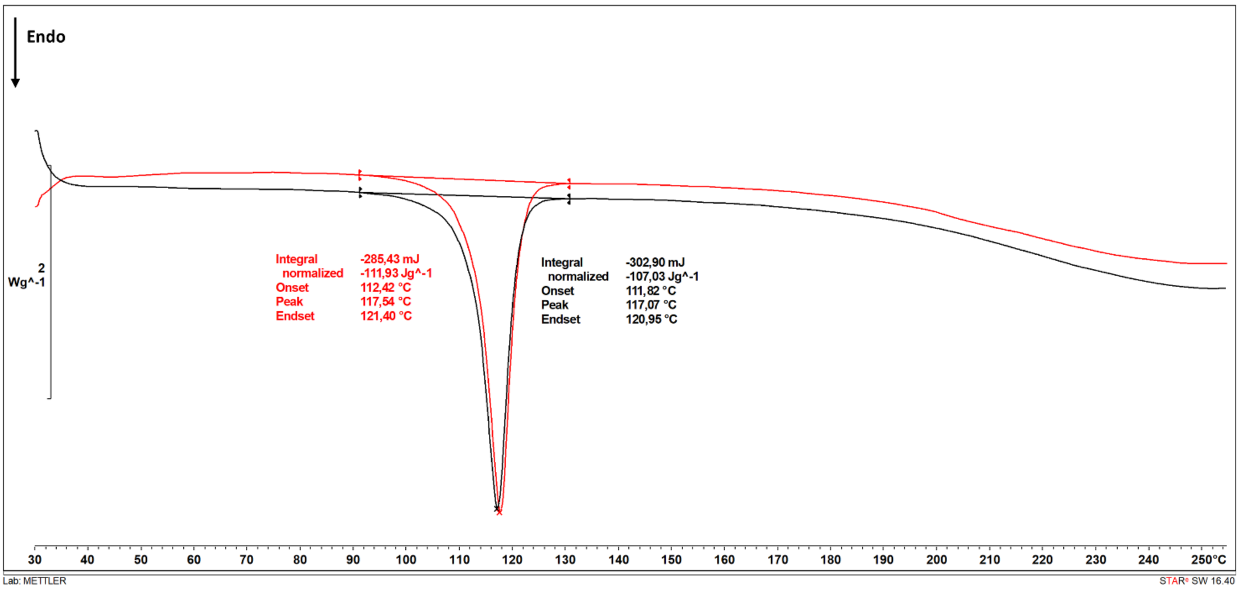

Figure S13. DSC thermograms of two different batches of the (**PEG-DME**)·(**caf**)$_{23}$·(**ana**)$_{23}$·(**6Fana**)$_{23}$ solid solution.

*4.7 Solid solution (**PEG-DME**)·(**caf**)$_{23}$·(**ana**)$_{17.25}$·(**6Fana**)$_{28.75}$*

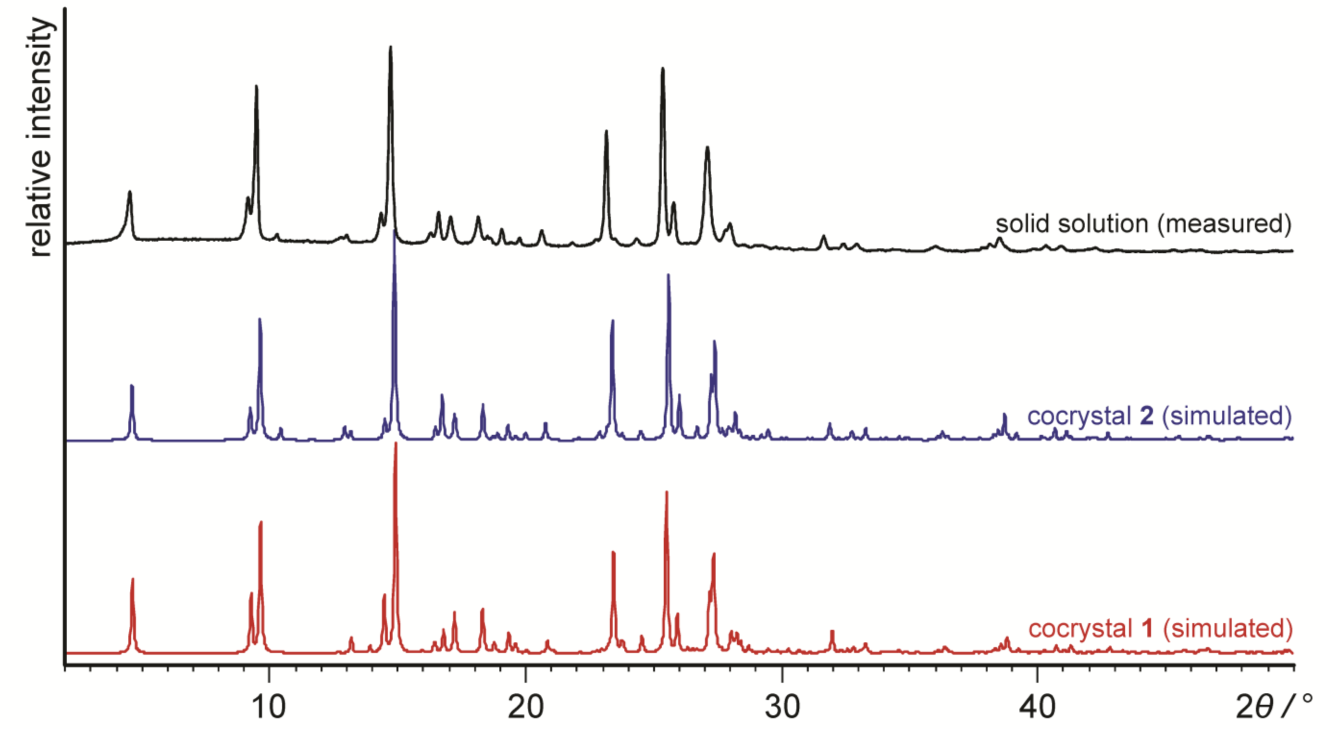

Figure S14. Measured diffractogram of the mechanochemically prepared (**PEG-DME**)·(**caf**)$_{23}$·(**ana**)$_{17.25}$·(**6Fana**)$_{28.75}$ cocrystal (shown in black), as compared to the calculated diffractogram of cocrystal **1** (derived from single crystal X-ray diffraction data, shown in red).

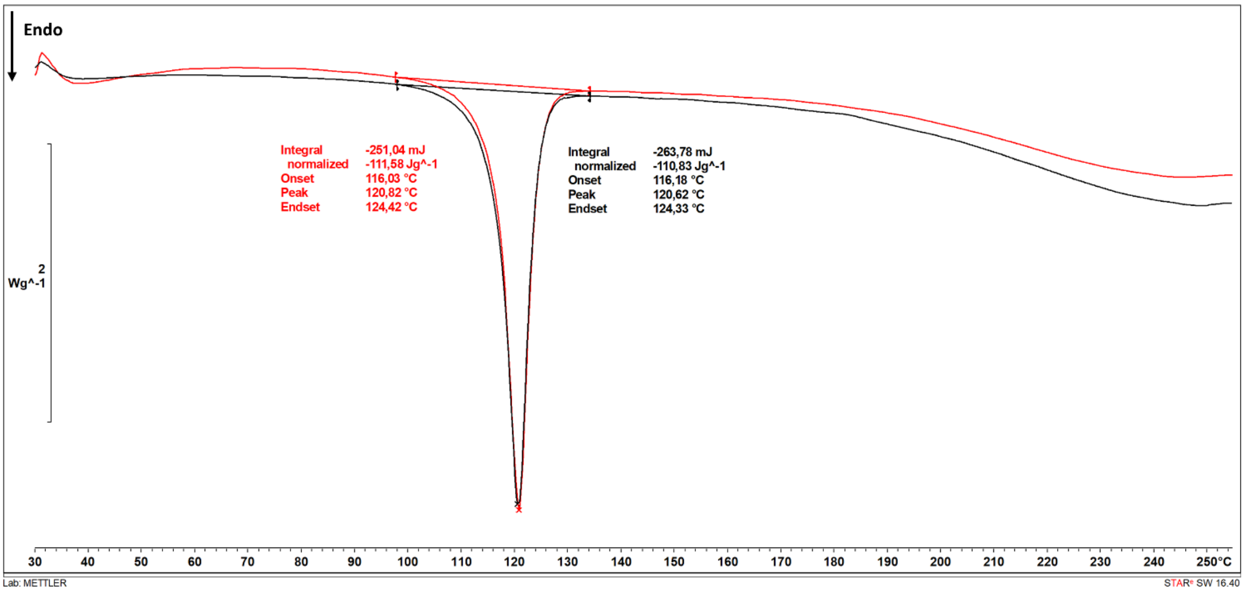

Figure S15. DSC thermograms of two different batches of the (**PEG-DME**)·(**caf**)$_{23}$·(**ana**)$_{17.25}$·(**6Fana**)$_{28.75}$ solid solution.

4.8 Solid solution (***PEG-DME***)*·*(***caf***)$_{23}$*·*(***ana***)$_{11.5}$*·*(***6Fana***)$_{34.5}$

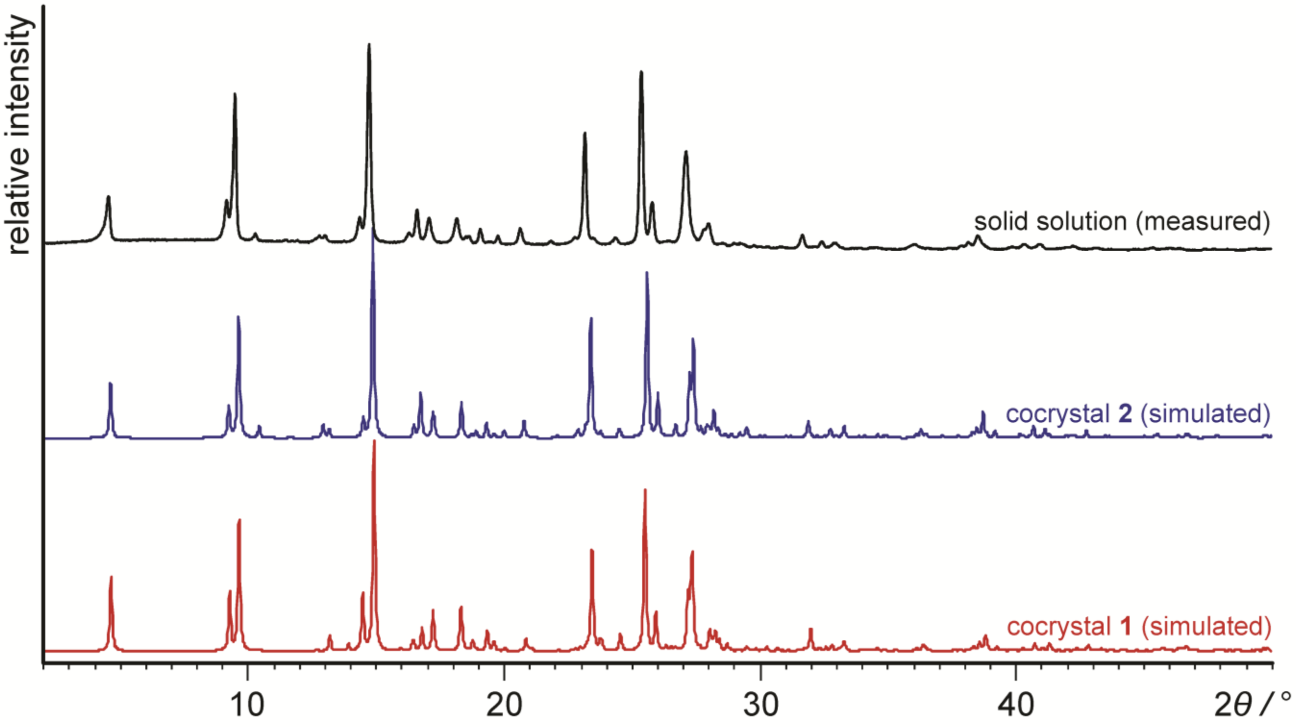

Figure S16. Measured diffractogram of the mechanochemically prepared (**PEG-DME**)·(**caf**)$_{23}$·(**ana**)$_{11.5}$·(**6Fana**)$_{34.5}$ cocrystal (shown in black), as compared to the calculated diffractogram of cocrystal **1** (derived from single crystal X-ray diffraction data, shown in red).

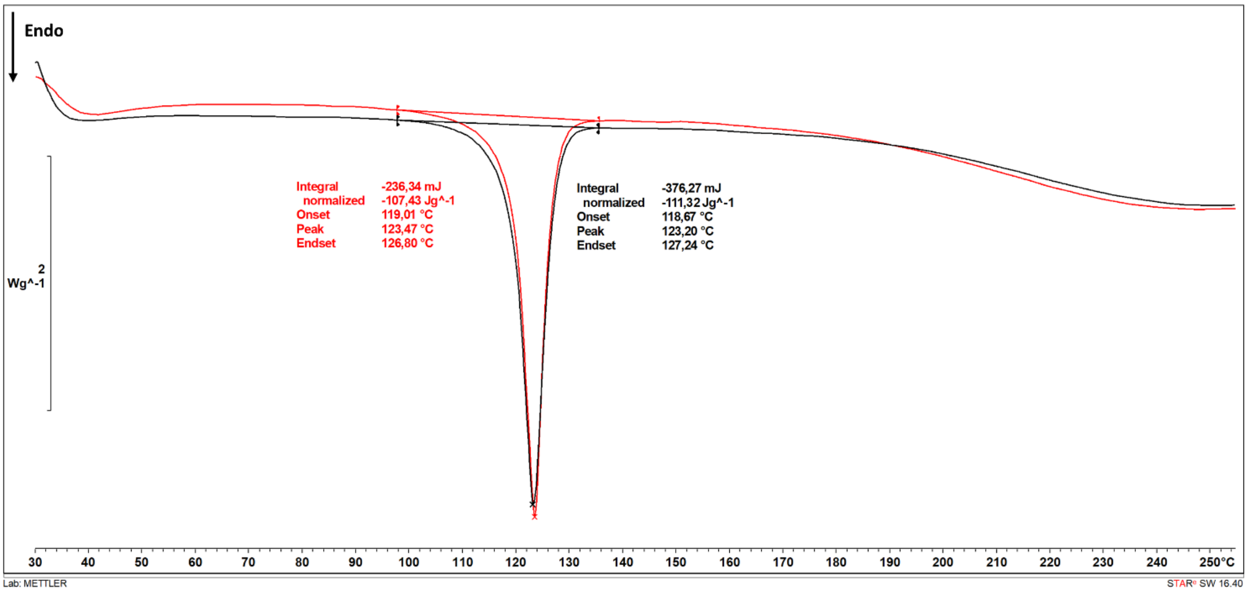

Figure S17. DSC thermograms of two different batches of the (**PEG-DME**)·(**caf**)$_{23}$·(**ana**)$_{11.5}$·(**6Fana**)$_{34.5}$ solid solution.

4.9 Solid solution (**PEG-DME**)·(**caf**)$_{23}$·(**ana**)$_{5.75}$·(**6Fana**)$_{40.25}$

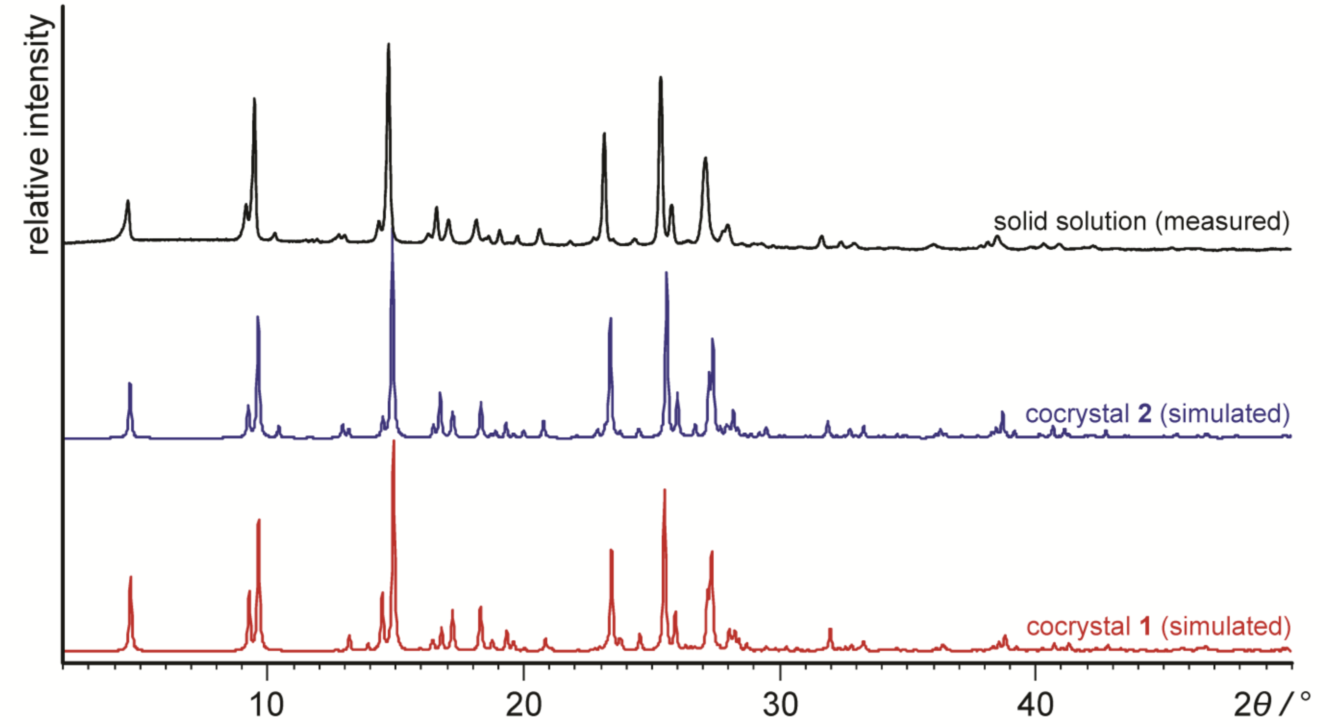

Figure S18. Measured diffractogram of the mechanochemically prepared (**PEG-DME**)·(**caf**)$_{23}$·(**ana**)$_{5.75}$·(**6Fana**)$_{40.25}$ cocrystal (shown in black), as compared to the calculated diffractogram of cocrystal **1** (derived from single crystal X-ray diffraction data, shown in red).

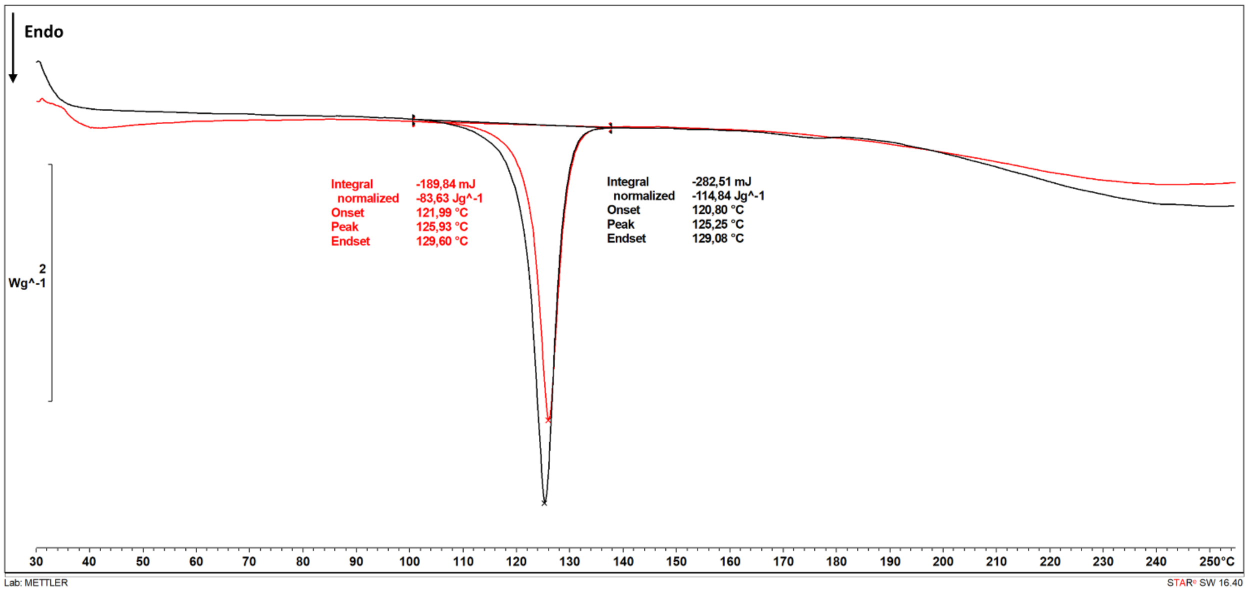

Figure S19. DSC thermograms of two different batches of the (**PEG-DME**)·(**caf**)$_{23}$·(**ana**)$_{5.75}$·(**6Fana**)$_{40.25}$ solid solution.

5. Preparation of single crystals of 1 and 2

Single crystals of **1** were obtained through melt crystallization using a mechanochemically prepared batch of **1**. Approximately 20 mg of **1** were placed in a glass vial and heated at 100 °C for 30 min. The material was subsequently cooled to room temperature at a cooling rate of 1 °C min^{-1} using in a bead bath (*LabArmor*™). The obtained single crystals were analyzed using PXRD before being subjected to single crystal X-ray diffraction studies.

Single crystals of cocrystal **2** were obtained through solution crystallization. About 10 mg of a mechanochemically prepared cocrystal batch were added to a 10 mL glass vial containing 4 mL of ethyl acetate. The solution was stirred for 120 min at room temperature and left to evaporate over the course of 72 hours.

6. Single crystal X-ray diffraction studies

The diffraction data for **1** and **2** were collected on a four-circle *Agilent SuperNova* (Dual Source) single crystal X-ray diffractometer using a micro-focus CuK_α X-ray beam (λ = 1.54184 Å) and a *HyPix-Arc 100°* hybrid pixel array detector. The sample temperatures were controlled with an *Oxford Instruments* cryojet. All investigated single crystals of **1** and **2** were poor scatterer.

All data were processed using the *CrysAlis^{Pro}* program.[6] Monoclinic and orthorhombic unit cell settings were evaluated for both **1** and **2** during data processing, structure solution and refinement procedures.

The crystal structures were solved with the *SHELXT* programme,[7] used within the *Olex2* software suite,[8] and refined by least squares on the basis of F^2 with the *SHELXL*[9] programme using the *ShelXle* graphical user interface.[10] All non-hydrogen atoms were refined anisotropically by the full-matrix least-squares method. Hydrogen atoms associated with carbon, nitrogen and oxygen atoms were refined isotropically [$U_{iso}(H_{C,N}) = 1.2U_{eq}(C,N)$; $U_{iso}(H_O) = 1.5U_{eq}(O)$] in geometrically constrained positions.

The F_o–F_c difference map of solids **1** and **2** suggested that one of the two anthranilic acid derivatives (**ana** and **6Fana**) in each asymmetric unit are disordered over two sites. The disorder was modelled using the SAME similarity restraint command in *SHELXL*.[9] The anisotropic parameters of the disordered **ana** and **6Fana** molecule were constrained using the SIMU and EADP commands in *SHELXL*.[9] Relevant crystallographic information and refinement parameters are shown in Table S3.

Table S3. Crystallographic information and details of refinement parameters of **1** and **2**.

	**1**	**2**
empirical formula	$C_{24}H_{28}N_6O_7$	$C_{24}H_{26}F_2N_6O_7$
M_r / g mol^{-1}	512.52	548.51
crystal system	monoclinic	orthorhombic
space group	$P2_1/c$	$Pna2_1$
a / Å	6.98249(19)	9.46228(13)
b / Å	38.1732(8)	38.3220(6)
c / Å	9.4519(2)	6.96023(12)
α / °	90	90
β / °	90.206(2)	90
γ / °	90	90
V / $Å^3$	2519.33(11)	2523.87(7)
Z	4	4
ρ_{calc} / g cm^{-3}	1.351	1.444
T / K	150.0(1)	150.0(1)
μ / mm^{-1}	0.848	1.004
F(000)	1080	1144
crystal size / mm^3	0.17 × 0.05 × 0.04	0.20 × 0.09 × 0.02
radiation	CuK_α (λ = 1.54184 Å)	CuK_α (λ = 1.54184 Å)
2θ range for data collection / °	4.633−66.590	4.615−77.623
index ranges	$-8 \le h \le 7$	$-9 \le h \le 11$
	$-45 \le k \le 39$	$-48 \le k \le 47$
	$-11 \le l \le 11$	$-8 \le l \le 7$
number of collected reflections	23044	24187
unique reflections	4437	5038
number of unique reflections	3758 [$I > 2\sigma(I)$]	4714 [$I > 2\sigma(I)$]
R_{int}	0.0431	0.0491
$R(F)$, $F > 2\sigma$(F)	0.0532	0.0440
$wR(F^2)$, $F > 2\sigma$(F)	0.1114	0.1123
$R(F)$, all data	0.0648	0.0469
$wR(F^2)$, all data	0.1160	0.1138
Δ_r (max., min.) e $Å^{-3}$	0.167/−0.204	0.180/−0.217
CCDC deposition number	2189953	2189954

7. High resolution TEM analyses

TEM experiments were devoted to study the morphology and the crystal properties of individual particles of **1** at atomic resolution. The study of individual particles required a TEM specimen containing a low density of particles to avoid their overlapping on the sustaining grid. The TEM specimens were prepared accordingly by depositing small amounts of pristine powders on a copper grid previously covered by a thin amorphous carbon film. The low atomic number of the chemical elements in **1**, the low density of particles on the carbon film and their small size resulted in a low contrast of the particles with respect to the amorphous carbon film. These features, together with the extreme sensitivity of this material to the damage (due to its the interaction with high energy electrons), make standard high resolution transmission electron microscopy (HRTEM) imaging on this kind of specimen impossible. Nevertheless, recent low dose approaches[11] enable imaging of radiation sensitive single particles at atomic resolution by a combination of in-line electron holography surveys and low dose and low dose rate HRTEM experiments.[12-13] The degree of sensitivity to electron irradiation is a function of the particle structure, orientation, and size. The microscope electron optical conditions used to acquire each image were tuned to vary the total density of electrons, ρ, in the $2 \leq \rho \leq 100$ $e^{-}Å^{-2}$ range. Low dose rate in-line holography surveys at a density of current of 0.2 $s{\cdot}e^{-}Å^{-2}$ were performed on thousands of particles in three different TEM specimens. About 500 HRTEM images were acquired and the relevant diffractograms were calculated and measured. A total of 56 particles were oriented to provide diffractograms with multiple nonequivalent lattice-spacing, thus enabling the calculation of the relevant zone axis and their simulation by full dynamical calculations.

TEM experiments were performed at room temperature using a *Jeol ARM UHR* TEM/STEM microscope operated at an acceleration voltage of 200 keV (electron wavelength of 2.5 pm) with a spatial resolution in HRTEM at optimum defocus of 0.19 nm.[14] The microscope was equipped with a corrector of the spherical aberration on the illumination system to enable a spatial resolution of 70 pm in scanning TEM (STEM).

The simulations of HRTEM micrographs and diffractograms were performed through full dynamical calculations on an octa-core CPU by *Java Electron Microscopy Simulations* (JEMS) program.[15] The crystallographic information file containing the crystal structure of **1**, derived from single crystal X-ray diffraction experiments, were used to index the diffractograms calculated from the HRTEM images of **1**.

Relevant HRTEM micrographs are shown in Figures S20-S22.

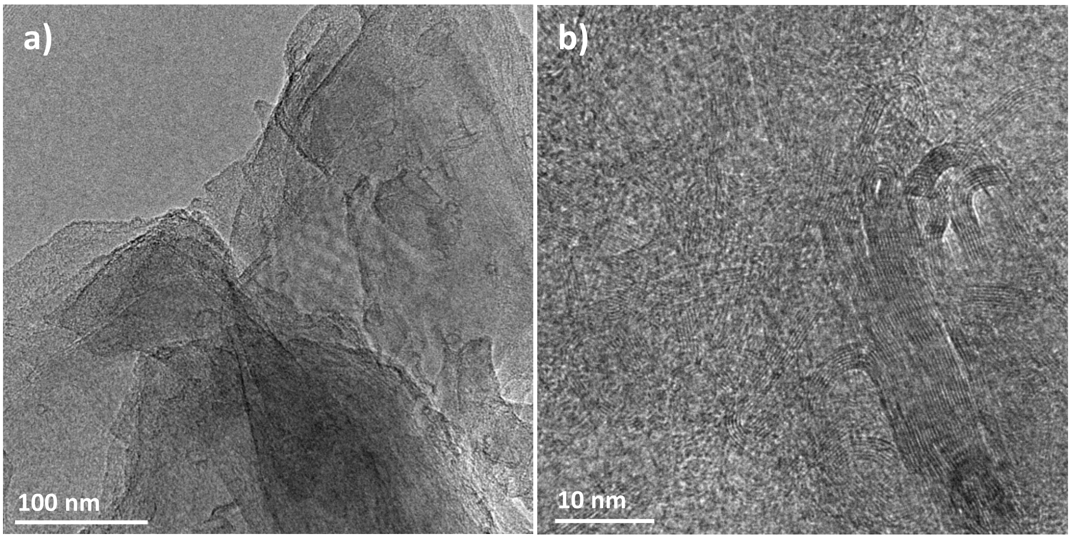

Figure S20. a) Part of an overlapped region of micrometric sized foil of **1**. b) HRTEM image zoomed on an area of micrometric fabric of **1**.

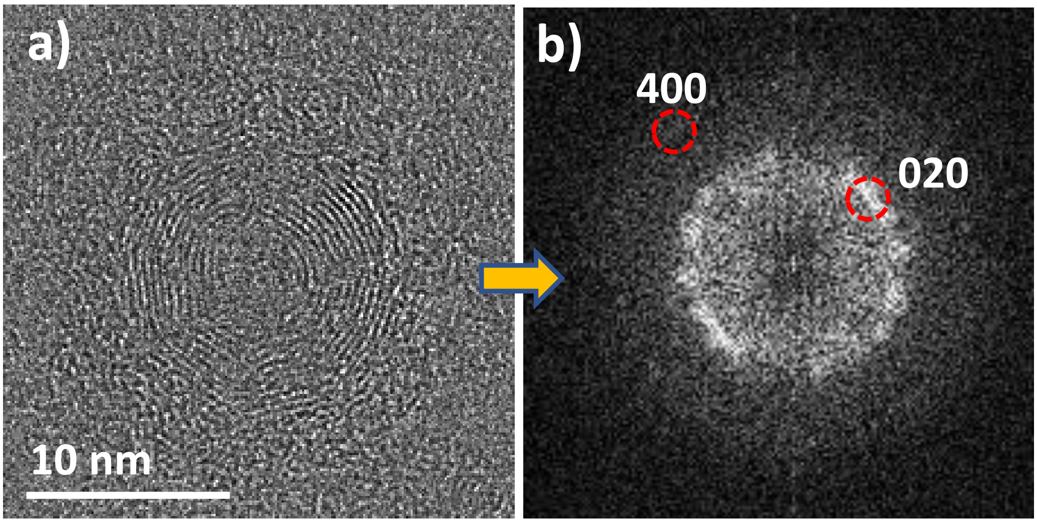

Figure S21. a) [001] zone axis HRTEM image of a particle made of **1**; b) diffractogram of a) together with the Miller's indices of some independent diffracted intensities.

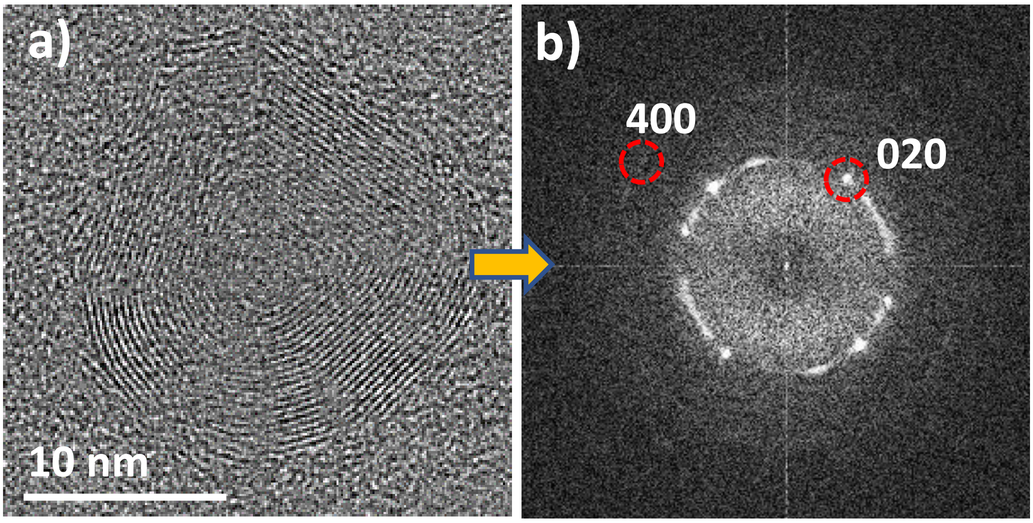

Figure S22. a) [001] zone axis HRTEM image of a particle made of **1**; b) diffractogram of a) together with the Miller's indices of some independent diffracted intensities.

9. Solid-form informatics

The crystal packing analysis was performed using the 'Crystal Packing Similarity' tool in the CCDC *Mercury* program (version 2022.3.0).[16] The analysis involved comparisons of clusters of 30 molecules, whereby 30% differences for intermolecular distances and 30° differences for intermolecular atomic angles were tolerated.[17] Molecular differences were allowed, while hydrogen positions were ignored in the analysis. The root-mean-square deviation value for the overlay of the matched 30 molecules in compared **1** and **2** was $RMSD_{30}$=0.121 Å (Figure 2d in the main text).

The CCDC *IsoStar* library of intermolecular interactions[18] and the *IsoStar* client (version 2022.3.0) were used to identify molecules suitable for isomorphic substitutions of the **caf** and **ana** components in cocrystal **1**. Fluoro-derivatives of **ana** were recognized as most promising after a visual inspection of the geometry of preferred intermolecular interactions between the following 'central groups' and 'contact groups':

1) central groups: aminophenyl, methylphenyl and halo-containing groups (bromophenyl, chlorophenyl, fluorophenyl, and more generally, aromatic bromo, chloro and fluoro)
2) contact groups: any alkyl and aromatic C−H groups, any C−F, C−Cl and C−Br groups, and uncharged $C(sp^2)/C(ar)-NH_2$.

References

[1] D. Hasa, G. Schneider Rauber, D. Voinovich, W. Jones, *Angew. Chem. Int. Ed.* **2015**, *54*, 7371–7375.
[2] N. Madusanka, M. D. Eddleston, M. Arhangelskis, W. Jones, *Acta. Cryst. B.* **2014**, *70*, 72–80.
[3] H. M. Rietveld, *Acta Cryst.* **1967**. *22*, 151–152.
[4] H. M. Rietveld, *J. Appl. Cryst.* **1969**, *2*, 65–71.
[5] A. A. Coelho, *J. Appl. Cryst.* **2018**, *51*, 210–218.
[6] *CrysAllisPro* 1.171.42.60a, Rigaku, 2022.
[7] G. M. Sheldrick, *Acta Cryst.* **2015**, *A64*, 3–8.
[8] O. V. Dolomanov, L. J. Bourhis, R. J. Gildea, J. A. K. Howard, H. Puschmann, *J. Appl. Cryst.* **2009**, *42*, 339–341.
[9] G. M. Sheldrick, *Acta Cryst.* **2015**, C*71*, 3–8.
[10] C. B. Hübschle, G. M. Sheldrick, B. Dittrich, *J. Appl. Cryst.* **2011**, *44*, 1281–1284.
[11] E. Carlino, *Materials* **2020,** *13*, 1413.
[12] D. Hasa, B. Perissutti, C. Cepek, S. Bhardwaj, E. Carlino, M. Grassi, S. Invernizzi, *Mol. Pharm.* **2013**, *10*, 211–224.
[13] D. Hasa, E. Carlino, W. Jones, *Cryst. Growth Des.* **2016**, *16*, 1772–1779.
[14] J. C. H. Spence, Experimental High-Resolution Electron Microscopy, 2nd ed.; Oxford University Press Inc.: New York, NY, USA, 1988; ISBN 0-19-505405-9.
[15] J EMS-S a A S. Version 4.3931U2021b20 by P. Stadelmann (https://www.jems-swiss.ch).
[16] C. F. Macrae, I. Sovago, S. J. Cottrell, P. T. A. Galek, P. McCabe, E. Pidcock, M. Platings, G. P. Shields, J. S. Stevens, M. Towler, P. A. Wood, *J. Appl. Cryst.*, **2020**, *53*, 226–235.
[17] P. A. Wood, M. A. Oliveira, A. Zink, M. B. Hickey, *CrystEngComm* **2012**, *14*, 2413–2421.
[18] I. J. Bruno, J. C. Cole, J. P. M. Lommerse, R. S. Rowland, R. Taylor, M. L. Verdonk, *J. Comput. Aided Mol. Des.* **1997**, *11*, 525–537.
